# Targeting gut microbiota for precision medicine: Focusing on the efficacy and toxicity of drugs

**DOI:** 10.7150/thno.47289

**Published:** 2020-09-14

**Authors:** Wuwen Feng, Juan Liu, Hui Ao, Shijun Yue, Cheng Peng

**Affiliations:** 1 School of Pharmacy, Chengdu University of Traditional Chinese Medicine, Chengdu, China.; 2 State Key Laboratory of Southwestern Chinese Medicine Resources, Chengdu University of Traditional Chinese Medicine, Chengdu, China.; 3 Shaanxi Collaborative Innovation Center of Chinese Medicinal Resources Industrialization, Shaanxi University of Chinese Medicine, Xi'an, China.

**Keywords:** gut microbiota, precision medicine, drug toxicity, drug efficacy, gut bacteria engineering

## Abstract

Intra- and interindividual variation in drug responses is one major reason for the failure of drug therapy, drug toxicity, and even the death of patients. Precision medicine, or personalized medicine, is a field of medicine that customizes an individual's medical diagnosis and treatment based on his/her genes, microbiomes, environments, etc. Over the past decade, a large number of studies have demonstrated that gut microbiota can modify the efficacy and toxicity of drugs, and the extent of the modification varies greatly from person to person because of the variability of the gut microbiota. Personalized manipulation of gut microbiota is an important approach to rectify the abnormal drug response. In this review, we aim to improve drug efficacy and reduce drug toxicity by combining precision medicine and gut microbiota. After describing the interactions between gut microbiota and xenobiotics, we discuss (1) the effects of gut microbiota on drug efficacy and toxicity and the corresponding mechanisms, (2) the variability of gut microbiota, which leads to variation in drug responses, (3) the biomarkers used for the patient stratification and treatment decisions before the use of drugs, and (4) the methods used for the personalized manipulation of gut microbiota to improve drug outcomes. Overall, we hope to improve the drug response by incorporating the knowledge of gut microbiota into clinical practice.

## Introduction

Drug responses vary among patients in terms of efficacy and toxicity, and this variation presents great challenges in optimizing drug therapy in the clinic 1. A surprising fact is that most drugs are effective in just 50-75% of patients, especially the drugs used for cancer chemotherapy, which are effective in only 25% of patients 2. In addition to the low effective drug response ratio, the alarmingly large number of toxic reactions confront us as well. For example, each year in the USA, 30,000-70,000 patients are hospitalized for acetaminophen toxicity 3,4. Thus, it is crucial to understand the mechanisms that drive the variation of drug efficacy and toxicity, and to apply suitable methods to improve drug outcomes. In light of this background, in 2015, the USA government launched the Precision Medicine Initiative, an initiative that aims to overcome individual variability and to make therapies more effective.

With the rapid progress in omics such as genomics and metabolomics, gut microbiota has become a focus in understanding the responses of human bodies to drugs. Gut microbiota is a complex group of microorganisms that dwell in the gastrointestinal tract in a mutualistic fashion with hosts. The intraindividual composition of gut microbiota is extremely dynamic, and the interindividual composition of gut microbiota varies greatly as well 5,6. Researchers have estimated that gut microbiota contains more than 1 × 10^15^ microbial cells and 5 × 10^6^ unique microbial genes, both of which outnumber human cells and genes 6. With these genes, gut microbiota can synthesize and release a series of enzymes with the capability to metabolize xenobiotics (chemical compounds that are not naturally present in host bodies such as environmental pollutants and drugs). The metabolic capability of gut microbiota is so strong that it is regarded as a virtual organ with metabolic capability exceeding that of the liver 7. When entering the gastrointestinal tract, orally administered drugs inevitably interact with gut microbiota 8,9. On the one hand, drugs can compositionally and functionally change gut microbiota; on the other hand, gut microbiota can chemically transform drugs 10. As a result, the pharmacological and toxic effects of drugs are highly influenced by gut microbiota. A sound understanding of these interactions enables us to manipulate gut microbiota to address the problems of drug efficacy and toxicity.

The term precision medicine is often used interchangeably with the terms personalized medicine, stratified medicine and person-centered medicine 11. Several articles have discussed gut microbiota and precision medicine 11-17. However, there has been a lack of systemic summarization of (1) the background information regarding why personalized manipulation of gut microbiota should be adopted to achieve improved drug responses and (2) the specific diagnostic biomarkers and therapeutic methods necessary to perform precision medicine using drugs. In this review, we aim to examine the use of precision medicine and gut microbiota for the clinical management of drug efficacy and toxicity. After describing the interactions between gut microbiota and xenobiotics, this review discusses the following aspects: (1) the effects of gut microbiota on efficacy and toxicity of drugs and the corresponding mechanisms, (2) the variability of gut microbiota, which leads to variation in drug responses, (3) the biomarkers used for patient stratification and treatment decision making before the use of drugs, and (4) the methods used to manipulate gut microbiota to perform precision medicine. In addition, we provide our perspectives on future studies.

## Xenobiotic metabolism system

### The fluxion of xenobiotics

When orally administered, the xenobiotics that have passed through the small intestine will ineluctably contact and thus chemically interact with gut microbiota. Gut microbiota synthesizes a battery of enzymes that are capable of transforming xenobiotics. These reactions include but are not limited to, rearrangement, oxidation, isomerization, esterification, condensation, reduction, and hydrolysis 9,10. After absorption, the transformed and prototype xenobiotics can be further metabolized by liver. The typical difference between liver metabolism and gut microbiota metabolism is that liver metabolism usually increases the molecular polarity and molecular weight of xenobiotics while the other one usually decreases the molecular polarity and molecular weight of xenobiotics 18. This can be explained by the fact that gut microbiota enzymes primarily catalyze hydrolytic and reductive reactions while liver enzymes typically perform oxidative and conjugative reactions 9. After intestinal absorption and/or liver metabolism, the prototype and transformed xenobiotics will be transported to other organs, discharged via the urine or released into the gut lumen via biliary excretion. The xenobiotics released by biliary excretion can undergo metabolism by gut microbiota again and be reabsorbed by enterocytes, and they can be metabolized by liver through enterohepatic circulation. Notably, the xenobiotics that are not orally administered can still undergo gut microbiota metabolism because of the biliary system and the efflux of intestinal epithelium.

### Complex interactions between xenobiotics and gut microbiota

**Figure 1:**
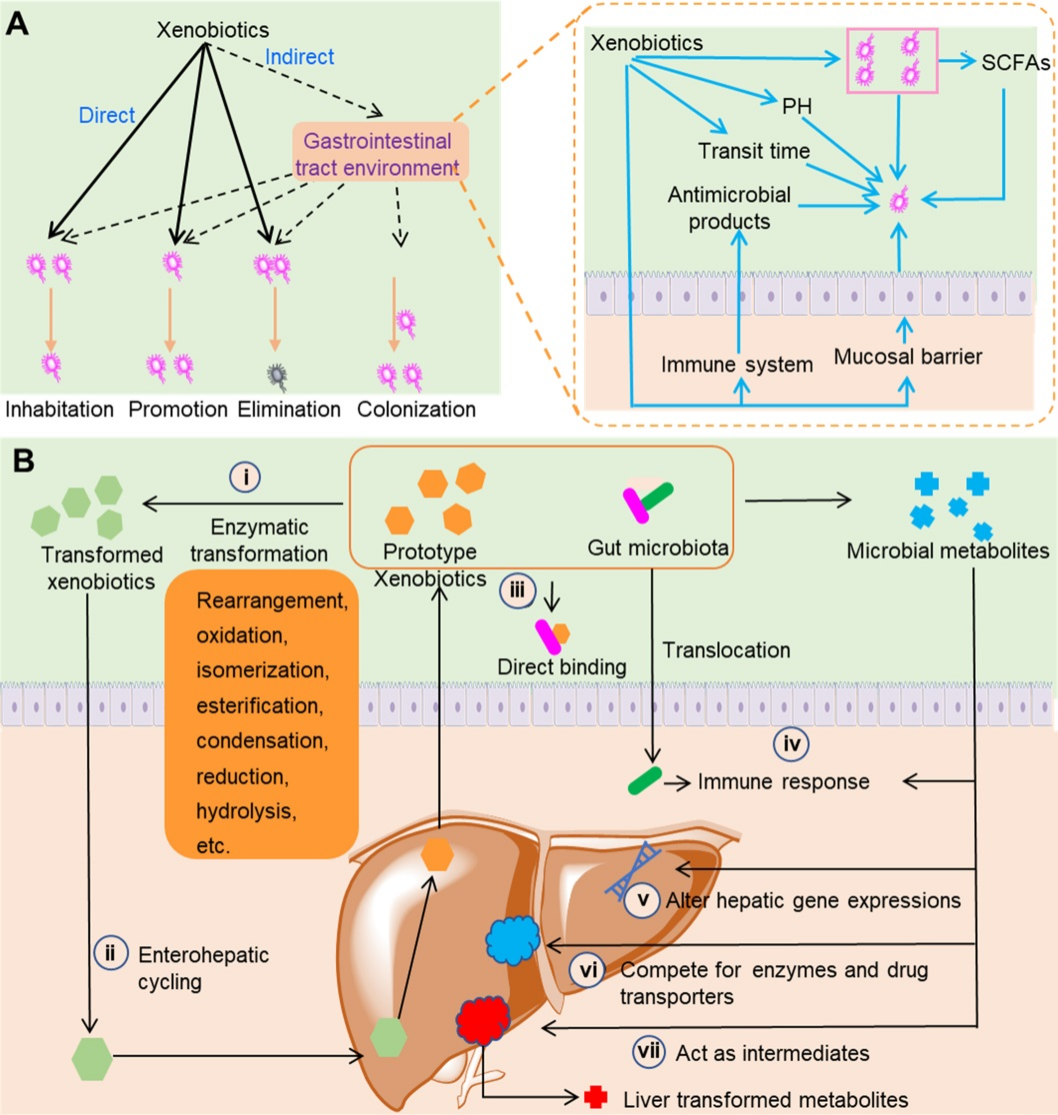
** Interactions between gut microbiota and xenobiotics.** (**A**) The effects of xenobiotics on gut microbiota composition. Xenobiotics can directly and indirectly change the composition of gut microbiota, including inhibition, promotion, elimination, and colonization. (**B**) Metabolism-related activity of the gut microbiota. Gut microbiota can synthesize and release enzymes with the ability to transform xenobiotics (i), and transformed xenobiotics may undergo further enterohepatic cycling (ii). Gut microbiota can directly bind to xenobiotics, which reduces the final absorption of xenobiotics by hosts (iii). When the intestinal epithelium is breached by xenobiotics or for other reasons, gut microbiota can trigger the immune response of hosts (iv). Gut microbiota can synthesize and release metabolites with the capability to modulate immune response of hosts (iv), alter hepatic gene expression (v), compete for enzymes and drug transporters (vi), and act as intermediates (vii).

Direct contact between xenobiotics and gut microbiota can lead to compositional changes in gut microbiota (Figure 1A). Xenobiotics can directly change the composition of gut microbiota via promotion, inhibition, and elimination. Prebiotics are nondigestible dietary compounds that are beneficial to hosts via selectively promoting the number or activity of certain groups of gut bacteria. Xenobiotics such as herbal polysaccharides and glycosides usually exhibit prebiotic-like effects as they can directly or indirectly provide carbohydrates as nutrients 19. While some xenobiotics can increase the number of certain gut bacteria, other xenobiotics such as antibiotics exhibit inhibitive or killing effects on gut bacteria. In addition to direct modulation, xenobiotics can modulate the gastrointestinal tract environment and the immune function of hosts to indirectly change the composition of gut microbiota. In gastrointestinal tract, the environmental pH and short-chain fatty acids (SCFAs) concentrations have a fundamental influence on the composition and metabolism of gut microbiota. For example, SCFAs can inhibit the growth of *Salmonella typhimurium* and *Salmonella enteritidis* at suitable concentrations and under the appropriate pH conditions 20. Xenobiotics such as polyphenols can affect the gastrointestinal pH and the levels of SCFAs to indirectly modulate the composition and metabolism of gut microbiota 21,22. The gastrointestinal transit time is a direct reflection of the time length of the interactions between xenobiotics and gut microbiota. It has been demonstrated that transit time can influence the overall composition, diversity, and metabolism of gut microbiota 23. Xenobiotics such as insoluble fibers can influence the intestinal transit time and thus indirectly change the composition of gut microbiota 24. Xenobiotics can also regulate the immune materials synthesized by hosts such as antimicrobial peptides and secretory immunoglobulin A. For example, aqueous extracts of the medicinal plant *Codonopsis pilosula* can increase the antimicrobial peptide levels in the Drosophila gut after sodium dodecyl sulfate-induced damage 25. Unlike the direct roles that only result in the promotion, inhibition, and elimination of bacteria, the indirect roles of xenobiotics can lead to the colonization of new bacteria 26.

Direct contact between xenobiotics and gut microbiota can also result in the transformation of xenobiotics and changes in gut microbiota metabolites (Figure 1B). Equipped with metabolic enzymes, gut microbiota can directly metabolize xenobiotics ranging from insoluble polysaccharides with high molecular weights to small molecule compounds such as flavones. Because of their combined transformation by gut microbiota and liver, some xenobiotics such as nonsteroidal anti-inflammatory drugs (NSAIDs) can undergo enterohepatic circulation 27. Gut microbiota can synthesize and release a large group of metabolites such as SCFAs, secondary bile acids, choline metabolites, and lipids 28,29. Xenobiotics can influence the production of gut microbiota metabolites. Some gut microbiota metabolites such as SCFAs can modulate the immune system of hosts to achieve the therapeutic effects of xenobiotics 30. Some of these gut microbiota metabolites can contend with xenobiotics for the host drug transporters and liver enzymes and thus influence the efficacy and toxicity of drugs. For example, the secondary bile acids derived from gut microbiota can contend with simvastatin for SLCO1B1 transporters, and correspondingly the plasma concentration and clinical efficacy of simvastatin are affected 31. The metabolism of xenobiotics by gut microbiota and the liver might function in tandem, as the gut microbiota can transform xenobiotics to intermediates that can undergo further liver metabolism. A typical example of this type of interaction was observed in a study of trimethylamine *N*-oxide (TMAO). Lipases in the small intestine and microbial glycyl radical enzymes in the colon sequentially metabolize dietary phosphatidylcholine and produce trimethylamine (TMA). Absorbed TMA can be further oxidized by flavin monooxygenases (FMO1 and FMO3) in the liver to produce TMAO, a compound that is responsible for an increased risk of cardiovascular diseases 32.

Xenobiotics can directly and indirectly interact with gut microbiota by direct binding and modulation of the host immune system (Figure 1B). *In vitro* incubation of *L*-dopa with *Helicobacter pylori* showed a conspicuous decrease of *L*-dopa, and *H. pylori* pre-incubated with *L*-dopa exhibited remarkably decreased adhesion to gastric epithelial cells 33. This study indicated that *L*-dopa can bind to the outer membrane proteins of* H. pylori*, and as a result the concentration of *L*-dopa in bacterial suspension was significantly decreased. The gastrointestinal epithelium has evolved to confine the gut microbiota and maintain the symbiotic relationship between the host and gut microbiota. Disruption of this architecture allows the infiltration of gut bacteria and the excess influx of materials such as lipopolysaccharides. The infiltration of gut bacteria and materials activates the immune systems of hosts, and finally leads to several diseases such as depressive disorder, cirrhosis and inflammatory bowel disease 34-36. Certain xenobiotics can disrupt the structure of gastrointestinal epithelium, leading to translocation of gut bacteria and activation of immune system. Heavy metal cadmium is an important environmental pollutant that has resulted in serious global issues. Exposure to cadmium induces an intestinal inflammatory response and gut architecture damage, which leads to an increase in gut permeability 37. The increase in gut permeability leads to bacterial translocation and an increase in blood endotoxin, which ultimately cause systemic inflammation.

## Nonnegligible role of gut microbiota in precision medicine: modulation of drug efficacy and toxicity

**Figure 2:**
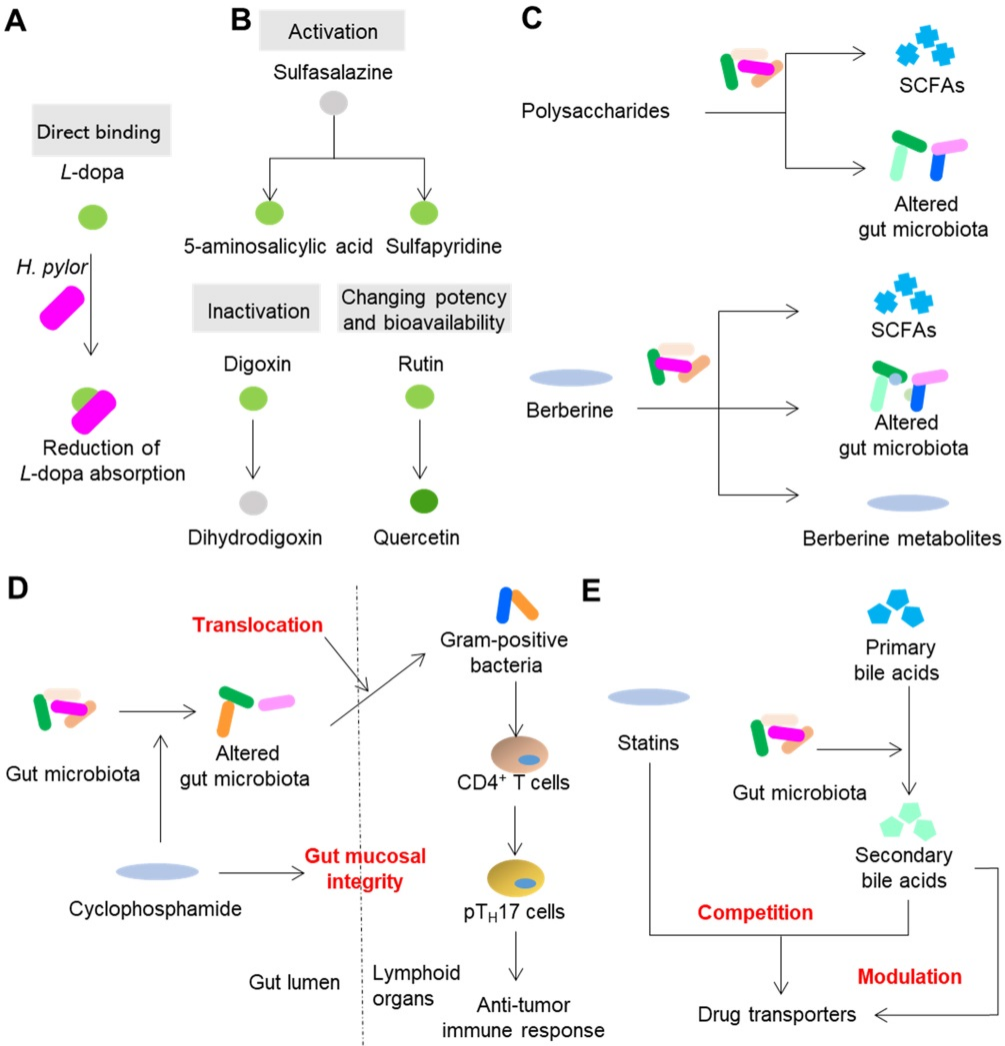
** Direct and indirect modification of drug efficacy by gut microbiota.** (**A**) Gut microbiota directly binds to drugs such as *L*-dopa. (**B**) Gut microbiota can inactivate, activate or change the potency and bioavailability of drugs via direct enzymatic metabolism. (**C**) Drugs modulate the composition and metabolism of gut microbiota to achieve efficacy. (**D**) Drugs modulate the spatial distribution of gut microbiota to achieve efficacy. (**E**) Gut microbiota synthesizes compounds with the ability to modulate and compete for drug transporters. Please see article text for the details on interactions and effects.

**Figure 3:**
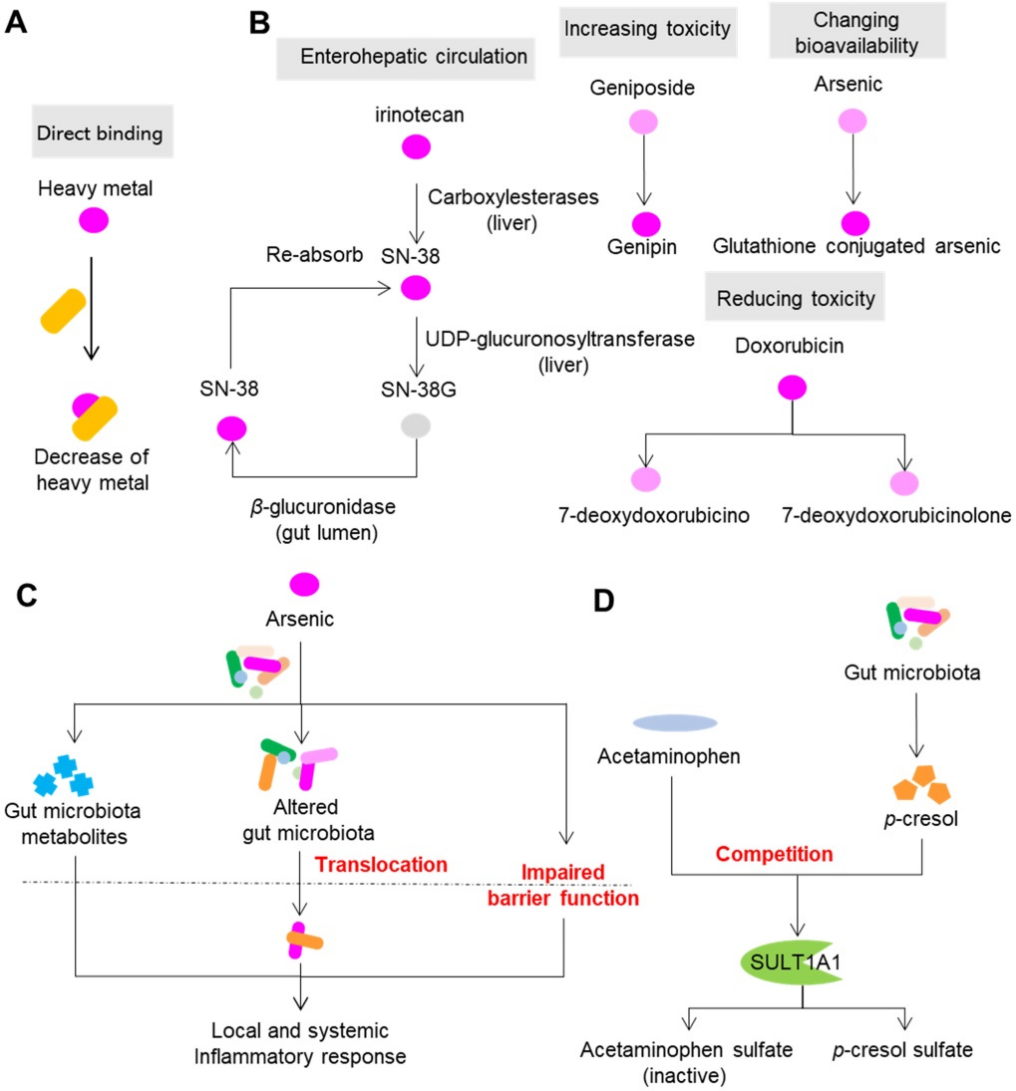
** Direct and indirect modification of drug toxicity by gut microbiota.** (**A**) Gut microbiota directly binds to drugs, especially heavy metal drugs. (**B**) Gut microbiota modulates the toxicity of drugs via direct enzymatic metabolism. (**C**) Drugs modulate the composition, metabolism and spatial distribution of gut microbiota to modulate toxicity. (**D**) Gut microbiota synthesizes compounds with the ability to compete for drug metabolism enzymes. Please see article text for the details on interactions and effects.

The impact of the direct and indirect interactions between gut microbiota and xenobiotics on the pharmacological and toxic effects of drugs is so profound that these interactions can cause the failure of drugs and even the death of patients. Therefore, to achieve precision medicine, it is necessary to understand the effects of gut microbiota on the efficacy and toxicity of drugs. Here, we discuss the effects of gut microbiota on drug efficacy and toxicity and the corresponding mechanisms. Because direct binding is the simplest type of interaction (Figure 2A, Figure 3A), we focus only on other types of reactions.

### Gut microbiota and the efficacy of drugs

Gut microbiota can inactivate, activate and change the potency and bioavailability of drugs via direct enzymatic metabolism (Figure 2B). Digoxin, a cardiac glycoside that was originally isolated from foxglove plants, is a typical example of a drug that can be inactivated by gut microbiota. Because of the strikingly narrow therapeutic range, the use of digoxin in clinic is a tough job. In approximately 10% of patients, a substantial amount of digoxin is converted into dihydrodigoxin, a cardio-inactive product 38. The conversion of digoxin by gut microbiota in patients can account for the inactivation of more than 50% of the administered digoxin 39, which significantly influences the bioavailability and clinical toxicity of this drug. Azo bonds, which can be reduced by azoreductases, are strategically used in drug development. Sulfasalazine, a prodrug with an azo bond, is widely used for ulcerative colitis and rheumatoid arthritis in clinic. Sulfasalazine can be detected in the feces of rats receiving antibiotics, while in the feces of animals raised under conventional conditions, it cannot be detected 40. Following oral administration, sulfasalazine is cleaved by azoreductases, which liberates two anti-inflammatory molecules 5-aminosalicylic acid and sulfapyridine. Gut microbiota can not only directly inactivate or activate drugs, but also change the potency and bioavailability of drugs, especially ethnomedicines. Flavonoids are a type of polyphenolic compounds that broadly exists in herbal medicines. Many flavonoid glycosides, such as rutin, hesperidin, and naringin, exhibit antiplatelet activity 41. These flavonoid glycosides can be transformed to aglycones by gut microbiota enzymes such as *β*-glucosidase and endo-*β*-glucosidase 42. The *in vitro* antiplatelet activities of these flavonoid aglycones such as quercetin, hesperetin and narigenin are stronger than those of their parental compounds 43. In addition to directly affecting the potency of flavonoids, the cleavage of sugar moieties from flavonoids can influence their bioavailability since the loss of sugar moieties undoubtedly affects lipophilicity and intestinal permeability 44. By direct enzymatic activation, inactivation, or alteration of the potency and bioavailability, the clinical efficacy of drugs is thus significantly affected (Figure 2B).

The normal composition, metabolism and spatial distribution of gut microbiota play indispensable roles in the homeostasis of hosts. Drugs, especially ethnomedicines, can change the composition, metabolism and spatial distribution of gut microbiota to (partly) achieve therapeutic effects especially those drugs that cannot be absorbed or exhibit poor bioavailability (Figure 2C). A type of compound that exists in all herbal medicines is polysaccharides. However, the human body cannot absorb or digest herbal polysaccharides due to the limited number of digestion enzymes encoded by human genome 19. On the contrary, gut microbiota encodes a series of enzymes that can degrade polysaccharides and yield metabolites that contribute to therapeutic effects such as SCFAs 30. Another way that polysaccharides achieve therapeutic effects is by modulating the composition of gut microbiota. For example, polysaccharides from *Hirsutella sinensis* mycelium exhibited anti-obesity effects on mice via selective promotion of the growth of *Parabacteroides goldsteinii*, and no alteration of SCFAs was observed 45. Berberine, an herbal compound with a bioavailability of less than 1%, exhibits powerful effects on insulin resistance. A recent animal study showed that berberine can alleviate insulin resistance by decreasing the synthesis of branched-chain amino acids (BCAAs) by gut microbiota 46. Gut microbiota is also responsible for the therapeutic effects of drugs that can be easily absorbed as prototypes. Metformin, a drug that belongs to the biguanide class, shows an absolute oral bioavailability of 50 to 60% 47. Compared with oral or intraduodenal delivery, intravenous delivery of metformin to human subjects is less effective in reducing blood glucose 47. In addition, the delayed release formulation of metformin showed blood glucose-lowering effects comparable to those of the standard formulation, even though the systemic level of metformin was significantly reduced 48. This evidence suggests that gut microbiota act non-negligible roles on therapeutic effects of metformin. Due to the progress of research, a series of recent studies have suggested that metformin can compositionally and functionally regulate gut microbiota to enhance the glucose-regulating effects of metformin. These effects include regulation of *Escherichia*, *Intestinibacter*, *Lactobacillus*, *Bacteroides fragilis*, *Akkermansia muciniphila*, *etc.*, and the ensuing effects of increased production of beneficial SCFAs, the alteration of the bile acid pool, the enhancement of the release of gut hormones glucagon-like peptide 1 and peptide YY, and secretion of peptide Amuc_1100, which can enhance barrier function and decrease endotoxemia 49. Translocation of gut microbiota can shape the immune response of hosts and thus further impact the efficacy of drugs. In the small intestine, the anticancer drug cyclophosphamide causes dysbiosis of mouse gut microbiota and induces the translocation of certain gram-positive bacteria into secondary lymphoid organs 50. These translocated bacteria then provoke the generation of “pathogenic” T helper 17 cells and memory T helper 1 immune response; thus, the therapeutic effect of cyclophosphamide is partly achieved (Figure 2D).

Competition and modulation of host genes and enzymes that are involved in transportation and metabolism of xenobiotics can also impact the efficacy of drugs (Figure 2E). Statins are *β*-hydroxy-*β*-methylglutaryl coenzyme A (HMG-CoA) reductase inhibitors that are used for the treatment of coronary artery disease. It has been reported that some patients are statin-resistant while others are statin-intolerant 51. Drug transporters such as SLCO2B1, SLC15A1, ABCC2, ABCB1, ABCG2 and ABCB11 are involved in liver uptake and hepatobiliary elimination of all statins 52. Notably, some of these transporters such as ABCC2 and ABCB1, also participate in the transportation of bile acids 53. Thus, it is hypothesized that the variation in the statin response can be attributed to the competition between bile acids and statins for the same transporters that participate in the uptake of statins. A few studies have supported this hypothesis. Animal studies have shown that statin treatment can cause gut dysbiosis and the expression of genes associated with bile acid transporters in the liver 54,55. In human patients, the response to simvastatin treatment is positively correlated with the levels of gut microbiota-produced secondary bile acids and the transporter SLCO1B1 31.

### Gut microbiota and the toxicity of drugs

Gut microbiota can modulate the toxicity of drugs via direct enzymatic metabolism (Figure 3B). In this process, the role of enterohepatic circulation has drawn much attention. Irinotecan (or CPT-11) is an intravenous drug that is extensively applied for colon cancer. However, more than 40% of patients receiving irinotecan can present with severe diarrhea, leading to dose reduction or termination of the drug 56. In the liver, CPT-11 undergoes a series of biotransformations: carboxylesterases first convert CPT-11 into the bioactive nuclear topoisomerase 1 enzyme inhibitor SN-38, and UDP-glucuronosyltransferase then converts SN-38 into inactive SN-38G 57. SN-38G is released into the gastrointestinal tract by the biliary tract, and then SN-38G is converted back into SN-38 by liberation of a sugar moiety by microbial *β*-glucuronidases. SN-38 in the intestine is toxic toward epithelial cells and is the cause of diarrhea in patients with colon cancer. *β*-glucuronidases also play a similar role in the toxic effects of NSAIDs 58. In the liver, NSAIDs first undergo glucuronidation and are then excreted into the intestine, where gut bacterial *β*-glucuronidases release the aglycones. The gut epithelial cells then absorb and metabolize the aglycones into reactive metabolites that are toxic to the intestinal wall.

Direct enzymatic metabolism can also influence toxicity by changing the toxicity and bioavailability of drugs. Doxorubicin is a widely used drug for carcinomas. However, its clinical use is limited due to its cumulative toxicity in normal tissues that can manifest as diarrhea, vomiting, hair loss and life-threatening cardiotoxicity 59. A recent study showed that doxorubicin can undergo deglycosylation by *Raoultella planticola*, and the products of deglycosylation exhibited reduced toxicity on model species *Caenorhabditis elegans* 60. Similarly, two human gut bacterial species, *Bifidobacterium longum* HY8001 and *B. fragilis*, can transform the herbal compound geniposide into genipin, a compound that shows increased toxicity toward HepG2 cells 61. By acetylation and esterification, human gut microbiota can transform toxic herbal compound aconitine into benzolyaconine and lipoaconitine, two compounds with reduced cardiotoxicity 62. Toxic heavy metals such as arsenic and mercury are commonly used in ethnomedicines to treat malignant diseases. Arsenic is widely used in ancient Chinese, Greek and Roman medicine to treat leukemia, syphilis and myeloma, and it can also cause cardiac disorders, respiratory failure, hypokalemia, and cerebral infarction 63,64. The toxicity of arsenic depends largely on its various states in mammals, including oxidoreductive states and methylation levels. *In vitro* and *in vivo* studies have demonstrated that gut microbiota can modulate the toxicity of arsenic via direct metabolism of arsenic (including oxidative methylation and glutathione conjugation), and this process also increases the bioavailability of arsenic 65,66. In addition to arsenic, it has been proven that gut microbiota can metabolize other heavy metals such as mercury and chromium, and thus change their bioavailability 67.

Drugs can change the composition, metabolism and spatial distribution of gut microbiota, which in turn affect the toxicity of drugs (Figure 3C). Methotrexate is a drug widely used for anticancer and immunosuppressive treatment; however, it can also cause gastrointestinal toxicity in the clinic. Methotrexate time-dependently changed the composition of gut microbiota in mice by inducing a significant decrease in *B. fragilis* and an increase in macrophage density 68. Gavage of mice with *B. fragilis* significantly decreased inflammatory reactions induced by methotrexate and restored the polarization of macrophages, suggesting the involvement of gut microbiota and host immune system in methotrexate-induced intestinal mucositis. In addition to direct metabolism induced by gut microbiota, heavy metals can cause dysbiosis and disruption of the gut barrier, which may further activate the host immune system 69. The study carried out by Lu *et al.* showed that ingestion of 10 ppm arsenic for four weeks significantly perturbed the composition of mouse gut microbiota and its metabolism various substances, such as bile acids and amino acids 70, suggesting that modulation of gut microbiota composition and metabolism are also associated with the toxicity of arsenic. Meanwhile, arsenic can disrupt the structure and function of intestinal epithelium, which enables the translocation of gut microbiota and increases endotoxemia, finally leading to local and systemic inflammatory response 71,72.

Competition with gut microbiota metabolites for host proteins and enzymes that participate in transportation and metabolism of xenobiotics can also impact the toxicity of drugs (Figure 3D). A typical example of this involves acetaminophen, a drug used worldwide to treat mild to moderate pain and to reduce fever. Severe and sometimes fatal hepatotoxicity is the major toxicity of acetaminophen, and the toxicity varies among individuals 73,74. The liver reduces the toxicity of acetaminophen by metabolizing it predominantly into the inactive compounds acetaminophen sulfate and acetaminophen glucuronide, and a small amount the of toxic compound *N*-acetyl-*p*-benzoquinone imine by CYP450 enzymes CYP2E1 and CYP3A4 75. *p*-Cresol is a microbial product produced during the metabolism of tyrosine and phenylalanine by gut microbiota. In the liver, *p*-cresol is metabolized to *p*-cresol sulfate, a substrate of the human cytosolic sulfotransferase 1A1 (SULT1A1), which is shared by acetaminophen 76. Individuals receiving high amounts of *p*-cresol sulfate exhibit reduced acetaminophen sulfate to acetaminophen glucuronide ratios in urine 77. Thus, the competition between *p*-cresol and acetaminophen hampers the capability of hosts to reduce the toxicity of acetaminophen by increasing the possibility of producing *N*-acetyl-*p*-benzoquinone imine.

### Gut microbiota in drug-drug interactions

Gut microbiota can also responsible for drug-drug interactions. The increasing antimicrobial drug resistance has become a serious global health issue, leading to an increase in infection-associated death. The use of drug combinations is encouraged to treat multidrug-resistant bacterial infections in the clinic; however, their effects on microbiota remain unexplored. Profiling of approximately 3,000 combinations of antibiotics, human-targeted drugs and food additives showed that 70% of drug-drug interactions are species-specific and 20% showed strain specificity, and antagonism is more common than synergism 78. In addition to antibiotics, the most striking example of drug-drug interactions involving gut microbiota is the co-administration of antiviral drug sorivudine with the antitumor drug 5-fluorouracil (5-FU) or 5-FU prodrugs, which has caused deaths and many serious side effects in Japan. Gut microbiota can transform sorivudine and into (*E*)-5-(2-bromovinyl) uracil (BVU). BVU is an inhibitor of liver dihydropyrimidine dehydrogenase, a catabolic enzyme that is responsible for detoxification of 5-FU. Thus, combined administration of sorivudine and 5-FU, increases the circulating 5-FU levels, leading to 5-FU-associated death 79,80. The combined use of different medicines is the essence of treatment with ethnomedicines such as traditional Chinese medicine. This strategy has been extensively adopted for thousands of years to achieve the goals of improved efficacy and reduction of side effects 81. By focusing on small molecules, a relatively large number of studies tried to explain the mechanism of the combinational use of ethnomedicines with the help of modern techniques such as network pharmacology and metabolomics 82,83. Polysaccharides ubiquitously exist in herbal medicines, yet their roles remain unknown because they cannot be directly absorbed. Using the ginseng decoction, a formula that mainly contains ginseng polysaccharides and ginsenosides, one study demonstrated that combinational use of ethnomedicinal compounds can facilitate the metabolism and absorption of bioactive compounds in the presence of gut microbiota 84. In that study, ginseng polysaccharides restored the perturbed gut microbiota composition in rats. In addition, ginseng polysaccharides significantly enhanced the bacterial metabolism and intestinal absorption of ginsenosides and promoted the growth of two ginsenoside metabolic bacteria including *Lactobacillus* spp. and *Bacteroides* spp. In addition to the combinational use of ethnomedicines, combinational use of chemical drugs and ethnomedicines is very common in China. Some studies have preliminarily demonstrated that gut microbiota is responsible for the efficacy-enhancing and toxicity-reducing effects of the combinational use of chemical drugs and ethnomedicines 85. Although the outcomes of the combination of chemical drugs and ethnomedicines are promising, their specific mechanisms remain to be resolved.

## Gut microbiota meets precision medicine: The variability of gut microbiota

Although the progress in understanding the roles of gut microbiota in the modulation of drug efficacy and toxicity has been striking, the extent of modulation still varies greatly person-to-person, leading to the unpredictable reactions to drugs, as evidenced by typical examples of the response of patients to PD-1/PD-L1 blockers 86. A metagenomic study of stool samples from patients with epithelial tumors revealed that the differences in the number of *A. muciniphila* are responsible for the variation in the therapeutic effects of PD-1 blockade 86. Dynamic analysis of patients with hepatocellular carcinoma receiving anti-PD-1 immunotherapy showed that *Proteobacteria* became predominant by the 12^th^ week in non-responders 87. Examination of the oral and gut bacteria of melanoma patients receiving PD-1 blockade showed relatively increased abundance of* Ruminococcaceae* in responding patients 88. Studies on berberine, NSAIDs, statins, and histamine-2 blockers have also demonstrated that gut microbiota shows great interindividual variability, and this variability contributes to the variability of drug response 89-92. Thus, to fulfill the aim of precision medicine, it is necessary to understand the variability of gut microbiota. Studies have demonstrated that the environment and host genes can shape the composition of gut microbiota 93, although there is controversy regarding which factor plays a more significant role 94,95. For infants, the mode of delivery (cesarean section or vaginal birth), type of feeding (formula-fed or breast-fed), gestational age, hospitalization, and drug use determine the composition of the gut microbiota 96. For adults, a large number of factors can shape the composition of gut microbiota, such as host genetics, age, the status of the host immune system, emotional stress, diet, and exercise 97. Due to the dynamic interactions between those factors and gut microbiota, the gut microbiota shows immense compositional variation between and within individuals 92,98.

The functions of gut microbiota include but are not limited to the metabolism of xenobiotics, biosynthesis of vitamins, steroid hormones, and neurotransmitters, modulation of host immune maturation, cell proliferation, vascularization and neurologic signaling 99. It is noteworthy that knowledge of the composition of gut microbiota does not necessarily translate into knowledge of its function as a large number of genes are expressed only under certain conditions. For example, expression of a CYP-dependent fatty acid hydroxylase-epoxidase from *Bacillus megaterium* ATCC 14581 is strongly inducible in the presence of phenobarbital 100. Although some studies have demonstrated that the function of gut microbiota tends to be much more stable in individuals in comparison with the composition of gut microbiota 98,101, evidence from other studies supports the opposite result, in that the function of gut microbiota at the metatranscriptomic (RNA) level is much more variable than that at the metagenomic (DNA) level 102. Another study using nontargeted, shotgun mass spectrometry-based whole community proteomics (metaproteomics) also showed that gut microbiota compositions and function at the protein level were different in different individuals 103. Since the composition of gut microbiota does not provide precise functional information, direct measurement of the function of gut microbiota is needed. Variations in the bioactivities of enzymes and the contents of metabolites are direct scalable manifestations of the functional variability of gut microbiota. The enzymes synthesized and released by gut microbiota play conspicuous roles in the metabolism of xenobiotics, and these enzymes show significant interspecies and interindividual variability. A recent study reported by the Human Microbiome Project gastrointestinal database clustered 3,013 microbial β-glucuronidase proteins into six groups, with each group exhibiting different functional capacities 104. This study also showed that β-glucuronidases exhibit distinct interindividual variabilities. Gut microbiota synthesize and release a series of metabolites that are helpful for the maintenance of health and harmful for the development of diseases 28,29. The contents of these metabolites such as SCFAs and choline have been demonstrated to vary greatly in different people 105,106.

**Figure 4:**
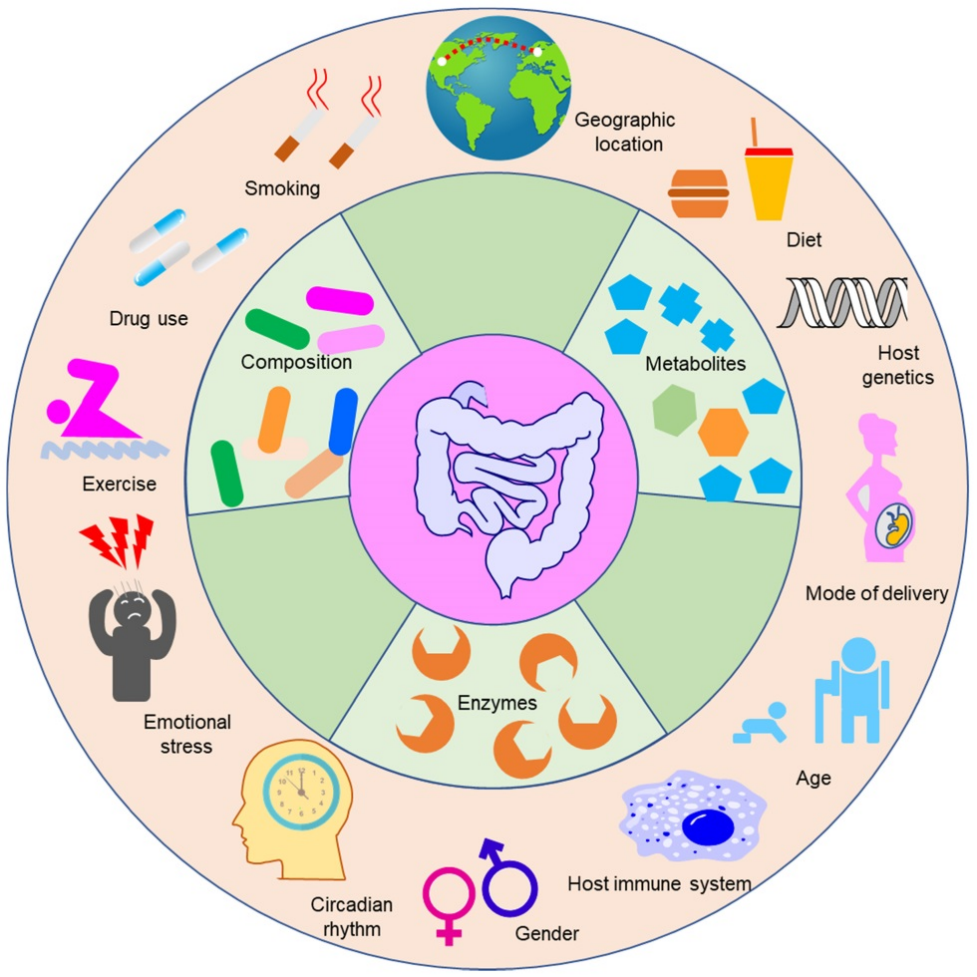
** The factors influencing the composition and function of gut microbiota.** Gut microbiota can modulate the efficacy and toxicity of drugs. However, the extent of modulation varies greatly from person to person and is highly dependent on the composition and function of gut microbiota of an individual. The composition and function of gut microbiota are influenced by multiple factors such as smoking, drug use, exercise, and emotional stress. The bioactivities of enzymes and metabolite contents are the direct scalable manifestations of the functional variability of gut microbiota.

The factors that shape the composition of gut microbiota can also impact the function of gut microbiota (Figure 4). For example, the age and geography of humans can influence the abundances of bacterial genes that code enzymes responsible for the metabolism of xenobiotics 107. Sex differences can influence the function of gut microbiota in mice exposed to arsenic 108. The circadian rhythm is an important factor that can impact not only the physiological function of mammals including immune responses, apoptosis, cell proliferation and differentiation, but also the efficacy and toxicity of drugs 109. Many studies have indicated that the circadian status of the host can affect the rhythmic fluctuations of gut microbiota in mice 110,111. Disruption of the normal gut microbiota rhythm induces genetic fluctuations in the intestine and liver, and hence gut microbiota can impact hepatic drug detoxification and hepatotoxicity 111. One typical example of this is liver toxicity induced by acetaminophen, which exhibited conspicuous diurnal variation 112,113. Administration of acetaminophen at night resulted in more severe liver damage compared with dosing in the morning. The mechanism of this phenomenon is linked to 1-phenyl-1,2-propanedione (PP), a gut microbial metabolite whose level in mice is significantly changed when light is present at the start of the resting period in comparison with the level when the light is absent at the start of the active period 114. PP can increase liver injury caused by acetaminophen when combined with acetaminophen, however, acetaminophen alone cannot lead to liver injury.

## Gut microbiota-associated markers for patient stratification and treatment decisions for the use of drugs

Currently, determination of the causation or association between bacterial species and diseases such as obesity and ulcerative colitis has allowed the development of gut microbiota-based biomarkers for precision disease diagnosis and risk stratification 16,115,116. Similarly, determination of the relationship between gut microbiota and drug outcomes will be helpful for screening biomarkers for personalized administration of drugs. Here, we discuss the possible gut microbiota-associated biomarkers for precision medicine in using drugs.

### Gut microbiota-based biomarkers

**Figure 5:**
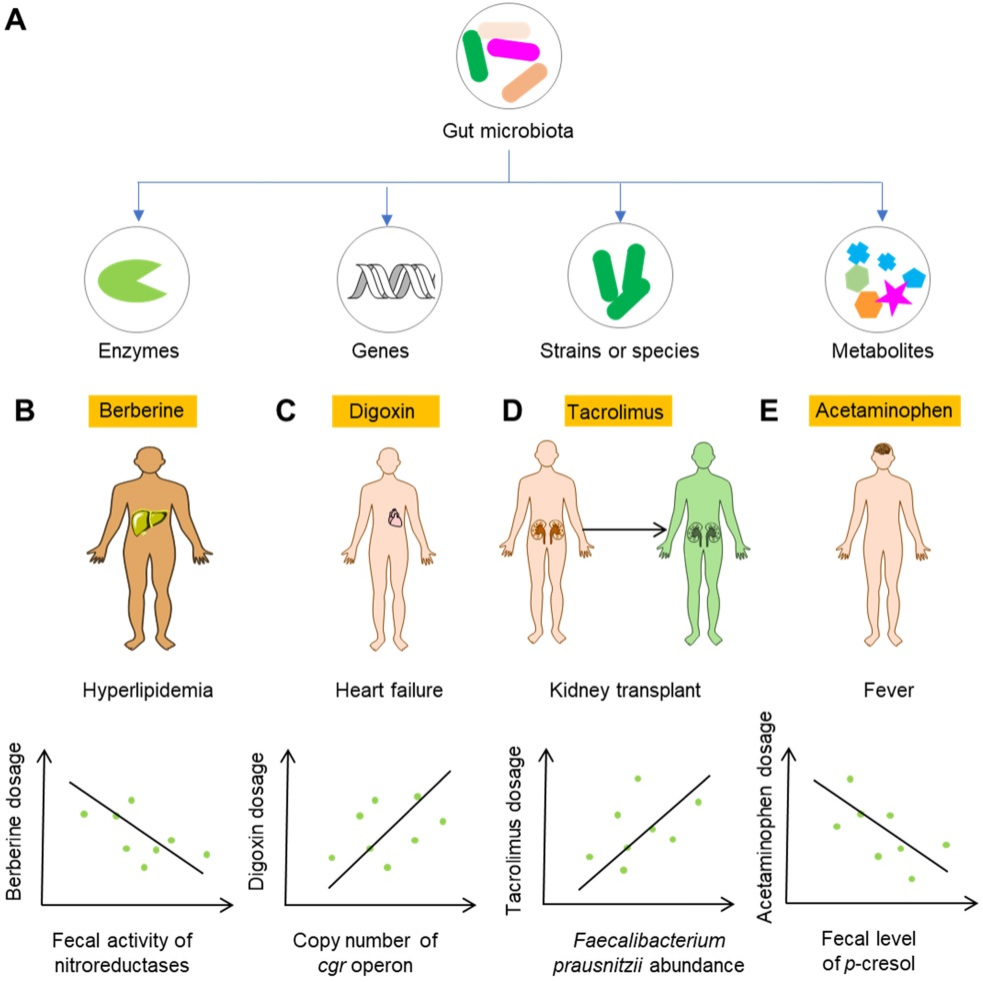
** The gut microbiota-based markers for the personalized use of drugs.** (**A**) Possible gut microbiota-based biomarkers for the personalized use of drugs, including genes, enzymes, bacterial strains or species, and gut microbiota metabolites. (**B**) The fecal activity of nitroreductases is positively correlated with the bioavailability of berberine in treating hyperlipidemia. Thus, the fecal activity of nitroreductases can serve as a biomarker for choosing the dosage of berberine. (**C**) *E. lenta* carrying the *cgr* operon can inactivate digoxin. Thus, the copy number of *cgr* operon can serve as a biomarker for choosing the dosage of digoxin or guiding physicians to distinguish patients who are likely to respond favorably to digoxin. (**D**) *F. prausnitzii* can transform tacrolimus into compounds with reduced potency. The abundance of *F. prausnitzii* is positively correlated with the need for an increased dosage in patients. (**E**) Gut microbiota metabolite *p*-cresol can compete with acetaminophen for liver enzymes, leading to increased toxicity. Thus, fecal *p*-cresol is a biomarker of a reduced dosage of acetaminophen.

Gut microbiota and gut microbiota-associated components including genes, enzymes, bacterial strains or species, and metabolites can be developed as biomarkers for the personalized use of drugs (Figure 5A). Perhaps the most feasible biomarker for precision medicine is the enzymatic activity of gut microbiota, as enzymatic reactions constitute a large proportion of the reactions leading to modification of drug efficacy and toxicity. Alternatively, analysis of the genes encoding these metabolomic enzymes can be used when detecting the enzymatic activiy is not easy. Berberine is an alkaloid separated from herbal plants such as *Berberis vulgaris* and *Coptis chinensis*. In China, berberine is extensively applied to treat bacteria-associated diarrhea and cardiovascular diseases such as hypercholesterolemia 117,118. Because the bioavailability of berberine is very low, it is usually used at a large dose. However, the use of a large dose of berberine in the clinic can also induce diarrhea, constipation, abdominal distension and abdominal pain 119,120. A recent study showed that nitroreductases, a group of enzymes from gut microbiota with the capability to catalyze the reductive reaction of drugs bearing nitroheterocyclic and nitroaromatic structures, convert berberine into the absorbable compound dihydroberberine and thus significantly improve the bioavailability of berberine in rats 121. Clinical studies have shown that absorption of berberine is positively associated with the gut bacterial activity of nitroreductases, highlighting the potential role of nitroreductase activity as a biomarker for the personalized use of berberine for the treatment of hyperlipidemia (Figure 5B) 122. Another example is digoxin. One study carried out decades ago showed that *Eggerthella lenta* in the gut is responsible for the conversion of active digoxin to cardio-inactive dihydrodigoxin 123. However, *E. lenta* cannot accurately predict the metabolism of digoxin in the gut, as *E. lenta*-colonized patients still show remarkable variation in dihydrodigoxin production 124. Recent studies showed that digoxin can induce the transcriptional activity of a cytochrome-encoding operon, and this “cardiac glycoside reductase” (*cgr*) operon is specific to drug-metabolizing strains of *E. lenta* 125,126. These studies suggest that the detection of genes associated with drug-metabolizing ability can be used for predicting the drug response (Figure 5C).

Although the abundance of a certain gut bacteria might not predict the variation of drug metabolism, as is the case for digoxin, it does not necessarily mean that the approach involving the detection of their abundance is of no use. Calcineurin inhibitors, such as the immunosuppressant tacrolimus, are widely used in kidney transplant recipients to reduce acute rejection rates and increase graft survival. Tacrolimus shows high variability of efficacy and toxicity in the clinic, which often leads to changes in dosage. However, sub-therapeutic dosages can result in immune rejection, and mega-therapeutic dosages can lead to nephrotoxicity and neurotoxicity 127. An analysis of kidney transplant recipients showed that the abundance of gut bacterium *Faecalibacterium prausnitzii* was positively correlated with the increase in tacrolimus dosing in recipients 128. A recent study revealed the role of *F. prausnitzii* in metabolizing tacrolimus into two different metabolites, and the major metabolite showed much less bioactivity *in vitro* 129. Thus, the abundance of *F. prausnitzii* may act as a biomarker for an increase in the dosage of tacrolimus (Figure 5D). The gut microbiota itself can synthesize a series of metabolites such as SCFAS, BCAAs, organic acids, and polyamines 28,29. These metabolites can serve as biomarkers for precision medicine as well. As discussed above, gut microbiota metabolite *p*-cresol can compete with acetaminophen for hepatic SULT1A1, and thus result in increased toxicity 130. Therefore, the level of *p*-cresol can act as a potential biomarker for the rational use of acetaminophen and other drugs utilizing the same metabolic enzymes in the liver (Figure 5E).

In recent years, the most active field involved in developing gut microbiota-based biomarkers for the prediction of drug responses has been immunotherapy. Typical examples are anti-PD-1 immunotherapy and anti-CTLA-4 immunotherapy. As mentioned in Section 4, the relative abundances of *A. muciniphila* and other bacterial species are associated with anti-PD-1 therapy outcomes 86,88. In addition, fecal microbiota transplantation (FMT) from individuals who responded to anti-PD-1 therapy to germ-free mice resulted in increased antitumor activity in comparison with that observed in mice receiving FMT from non-responder donors. These studies demonstrated that *A. muciniphila* and other bacteria can serve as biomarkers for predicting the outcomes of PD-1 blockade in patients with epithelial tumors and metastatic melanoma 86,88,131. Another typical example related to immunotherapy is CTLA-4 inhibitors. Ipilimumab, an important monoclonal antibody used for the treatment of metastatic melanoma that blocks the CTLA-4, induces typical toxicities, including intestinal inflammation and even the death of recipients, because of dysregulated mucosal immunity 132. Using next-generation metagenomic sequencing, Dubin *et al.* found out that a higher abundance of *Bacteroidetes* is correlated with ipilimumab-induced colitis in humans 133. Vétizou *et al.* found that T cell responses in mice and patients specific for *Bacteroidetes thetaiotaomicron* or *B. fragilis* were responsible for the efficacy of CTLA-4 blockade 134. A similar study showed that the baseline abundances of *Faecalibacterium* and other gut bacteria were correlated with the efficacy of ipilimumab and the risk of colitis caused by ipilimumab 135. These three studies demonstrated that *Bacteroidetes*,* B. fragilis* and other bacterial species can serve as biomarkers for the efficacy and toxicity of CTLA-4 inhibition.

### Emerging integrative approach

Currently, researchers studying precision medicine have placed a major emphasis on the variability of human body, and thus, the variability of gut microbiota is often neglected. For example, the precision oncology and anti-TNF agents used for treatment of Crohn's disease have not been proven to work well 136,137, and the omission of gut microbiota might be the reason for the failure. In contrast, focusing mainly on gut microbiota can lead to the failure of the personalized use of drugs as well considering the significant impact of hosts on drug toxicity and efficacy. Therefore, integration of the variabilities of gut microbiota and hosts is needed, especially for drugs that can undergo combined metabolism by gut microbiota and hosts. For example, the widely prescribed anticancer drug CPT-11 shows significant interindividual pharmacokinetic variability 138, and both human body and gut microbiota are believed to be responsible for this variation. In the liver, the variants responsible for liver metabolism of CPT-11 such as UGT1A1*28 and UGT1A7-57T/G, can lead to significant variability in the toxicity of CPT-11 139. In the gastrointestinal tract, the capability of gut microbiota to reactivate the excreted, inactive metabolite of CPT-11, also shows great interindividual variability 140. Similarly, the widely used anticoagulant drug warfarin can undergo significant variation in both human and microbiota aspects. VKORC1 (vitamin K epoxide reductase) and cytochrome CYP2C9 polymorphisms can cause great interindividual variation in terms of drug clearance and drug sensitivity 141. In addition, gut microbiota and diet also impact interindividual warfarin metabolism differences by interfering with the production of vitamin K, a compound responsible for the synthesis of blood coagulants 142-144. Therefore, only focusing on gut microbiota or hosts will omit the contribution of other factors to the interindividual variation of drug toxicity and efficacy. Correspondingly, for drugs that can be metabolized by both gut microbiota and the human body, integration of gut microbiota and host metabolism is needed to predict the variation of drug responses and final drug outcomes.

To achieve the aim of precision medicine, it is needed to separate and quantify the contribution of gut microbiota to the *in vivo* toxicity and efficacy. However, the gut microbiota and hosts are so highly intertwined that the separation of the contribution of hosts and gut microbiota to final drug outcomes is incredibly challenging, especially in the case where the host and gut microbiota can transform drugs into the same metabolites. Fortunately, with the development of germ-free and gnotobiotic animal models and new algorithms, it is now possible to achieve this aim. Brivudine is an oral antiviral drug that belongs to nucleoside analog and can be metabolized into bromovinyluracil by the host and gut microbiota. By using brivudine as the model drug, Zimmermann *et al.* established an approach to disentangle the contribution of the host and gut microbiota to drug metabolism in mice 145. To measure the extent of the metabolism of brivudine across tissues, they detected the contents of brivudine and bromovinyluracil in various locations including feces, serum, cecum, thymine, and liver in conventional, germ-free and gnotobiotic mice. They also built a pharmacokinetic tool to quantitatively estimate the contribution of gut microbiota to systemic brivudine and bromovinyluracil exposure based on the bioavailability, the absorption of drug and drug metabolite, the drug-metabolizing activity of host and gut microbiota, and the intestinal transit kinetics. The authors also used benzodiazepine clonazepam to examine the general applicability of the method. In another study, they expanded their model to examine host glucuronyltransferase activity and cecum bacterial deglucuronidation and tested the effects of the enterohepatic circulation on systemic drug metabolite exposure 146. These studies offer us excellent experimental and computational strategies to determine the contribution of gut microbiota to drug metabolism.

**Figure 6:**
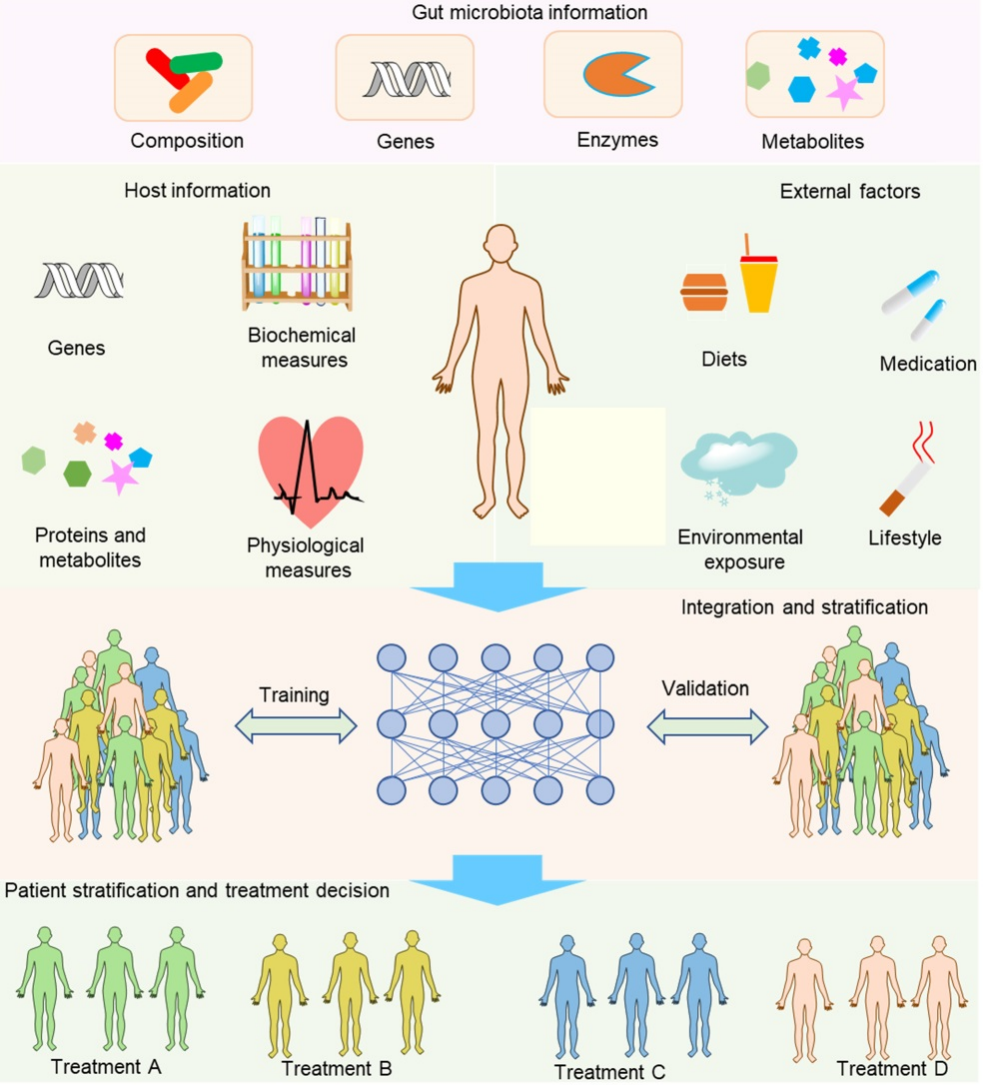
** Integration of multiple factors to develop integrative biomarkers for patient stratification and treatment decisions.** The parameters used for establishing integrative biomarkers can be derived from hosts, gut microbiota and other external factors. When an integrative biomarker has been preliminarily screened, a further validation step is needed to confirm the suitability. The major characteristic of integrative biomarkers in comparison with conventional biomarkers is that an integrative biomarker comprises multiple variables that act holistically to influence drug outcomes.

Compared with separating the contribution of hosts and gut microbiota to the drug metabolism, integrating information about hosts, gut microbiota and other external factors to develop integrative diagnostic biomarkers is far more important and challenging to achieve the aim of precision medicine (Figure 6). Host information may include genetic and physiological factors such as the polymorphism and activity of hepatic microsomal enzymes, blood pressure, and the functional capability of the immune system. The gut microbiota information may include compositional, genetic, enzymatic information and metabolites. Sometimes, more comprehensive information might be needed, such as information about demographics, family history, and lifestyle. To gather the information, we need many methods and models such as whole-genome shotgun sequencing, nuclear magnetic resonance (NMR) and/or mass spectrometry (MS)-based metabolomics, activity-based and probe-enabled proteomics, culturomics, germ-free and gnotobiotic animal models, and gut-on-a-chip systems 147-149. To achieve the aim of biomarker discovery, several sophisticated methods are needed to parse out the associations with diseases, for instance, the complex biostatistics, or artificial intelligence algorithms 12. In contrast to traditional biomarkers that only contain one factor for each biomarker, an integrative biomarker can constitute several factors such as the diet, polymorphism of host genes and gut microbiota composition. For example, vedoNet, a neural network algorithm that incorporates the gut microbiota and clinical data, can predict patients' response to inflammatory bowel disease treatment 150. When a possible integrative biomarker has been preliminarily screened, a further validation step is needed to confirm the biomarker. In addition, despite the promising future of integrative biomarkers, the process needed to discover integrative biomarkers is rather challenging because of the lack of integrative tools to reveal the complex interactions of host, gut microbiota, and external factors. Even so, we anticipate that more and more tools will be developed to overcome the obstacles of this inchoate approach. For example, Noronha *et al.* recently integrated human metabolism, gut microbiome, disease, and nutritional information to build the Virtual Metabolic Human database, enabling a series of works such as predicting the metabolism of human and gut bacteria not only in healthy but also in diseased states 151.

## Gut microbiota-oriented precision medicine

**Figure 7:**
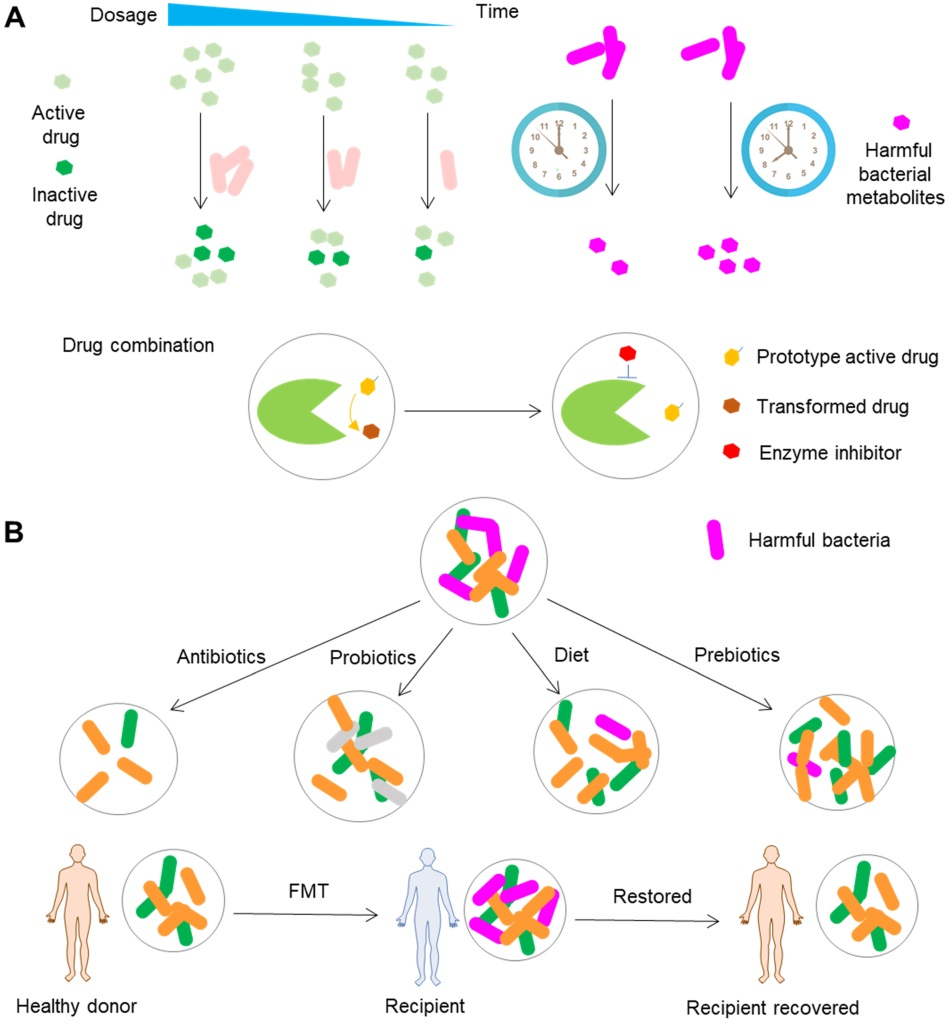
** Conventional gut microbiota-oriented approaches for precision medicine.** (**A**) Rational use of drugs, including the selection of a suitable dosage and drug administration time and the combinational use of (non-antibiotic) drugs. A dosage based on the levels of drug-metabolizing bacteria can ensure the consistency of the levels of active drugs between and within individuals. At different time points, gut microbiota release different levels of metabolites that can act synergically to enhance the toxicity of drugs such as acetaminophen. The combined use of drugs especially bacterial enzyme inhibitors can inhibit the metabolizing effects of gut microbiota on drugs. (**B**) Conventional methods for modulating gut microbiota. Gut microbiota can be modified by antibiotics, probiotics, diet, prebiotics and FMT. Notably, the extent of modification is not precise.

**Figure 8:**
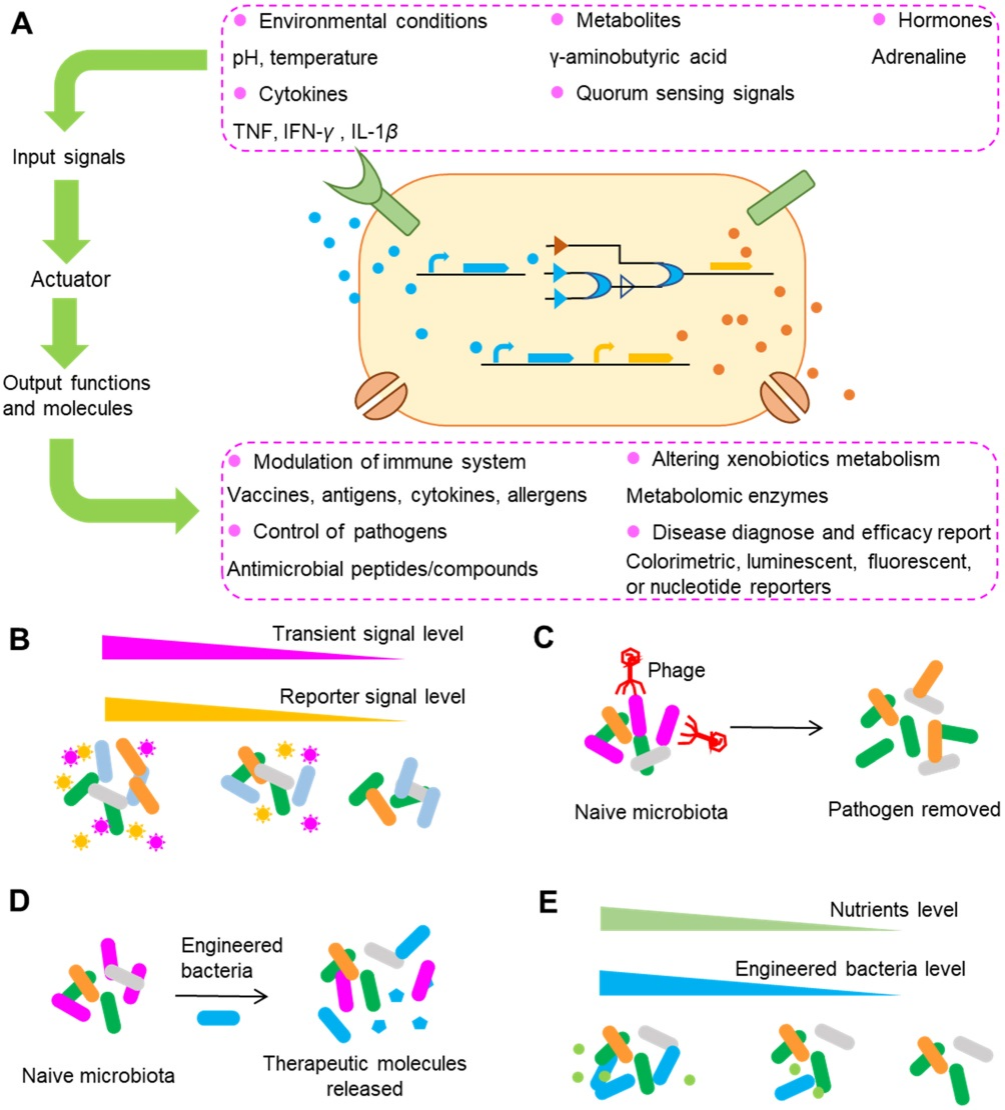
** Typical strategies used in engineering gut bacteria for precision medicine.** (**A**) An overview of engineered bacteria used for sensing and responding to surrounding signals. After sensing surrounding signals such as pH and temperature, engineered bacteria can synthesize and release a series of molecules that can modulate the immune system, control pathogens, alter xenobiotic metabolism, *etc.* (**B**) Engineering gut bacteria to report transient molecules that can be easily degraded, absorbed or modified in the gut as surrogate biomarkers. (**C**) Precise depletion of gut bacteria by engineered phages. The specific targets for depletion can be undesirable genes or polymorphisms encoding drug metabolism enzymes. (**D**) Bacteria can be conferred with the ability to release therapeutic molecules or other abilities such as the ability to modulate the metabolism of drugs. (**E**) Biocontainment of engineered bacteria exemplified by auxotrophy, in which the number of engineered bacteria can be regulated by the nutrients supplied. Using these strategies alone or in combination, the level of drug metabolized by gut microbiota can be precisely regulated and thus the final drug response can be controlled.

Currently, the manipulation of gut microbiota, especially the precise editing of gut microbiota, is a favorable approach for the treatment of diseases. For example, supplementation with probiotics and prebiotics has been used to treat gut microbiota-associated diseases such as ulcerative colitis and diarrhea 152-154. More recently, precise editing of gut microbiota by tungstate has shown satisfactory efficacy in animal colitis models by prevention of dysbiotic increase of *Enterobacteriaceae* family during intestinal inflammation 155. Although there are a great number of studies reporting disease treatment by manipulating gut microbiota alone, research on manipulating gut microbiota to improve drug outcomes are still in an early state 156. Here, focusing on gut microbiota, we discuss possible methods for precision medicine in using drugs (Figure 7 and Figure 8).

### Drug usage

The most important road toward precision medicine can be achieved by rational use of drugs (Figure 7A). There is no doubt that precisely modifying the dosage according to the metabolomic capability of gut microbiota is the first choice. The time for the administration of drugs should be taken into consideration as well, since the circadian rhythm plays an important role in the compositional and functional fluctuations of gut microbiota and can significantly impact the toxicity of drugs by modulation of gut microbiota metabolites as exemplified by the liver toxicity of acetaminophen in mice 114. Targeting gut microbiota by combinational use of drugs is a good alternative as well, especially the combinational use of drugs targeting bacterial enzymes responsible for the transformation of drugs.

Among a plethora of gut microbiota enzymes, *β*-glucuronidases are definitely the most striking enzymes because of their conspicuous functions in changing the bioavailability, efficacy and toxicity of drugs. The development of bacterial *β*-glucuronidase inhibitors is thus an intriguing approach to achieve improved efficacy and reduced toxicity 157,158. *β*-glucuronidases are a group of enzymes that are extensively distributed in gut bacteria and are expressed in the human liver 159,160. Correspondingly, the ideal inhibitor should be selective for bacterial *β*-glucuronidases. Cheng *et al.* found a potent *E. coli β*-glucuronidase-specific inhibitor TCH-3562 that shows no activity toward human *β*-glucuronidase 161. Coadministration of CPT-11 and TCH-3562 did not impact the active SN-38 content in mice, suggesting that TCH-3562 may be a promising agent to prevent CPT-11-induced diarrhea. The intake of diclofenac, an NSAID, is associated with increased anastomotic leakage after intestinal and colorectal surgery 162. Microbial conversion of diclofenac metabolites released into the gastrointestinal tract by *β*-glucuronidases brings harmful effects on intestinal mucosa. A recent study showed that combinational use of the microbial glucuronidase inhibitor Inh1 can significantly decrease the severity of anastomotic leakage caused by diclofenac 163. Regorafenib is a multikinase inhibitor with excellent antitumor effects. Microbial reactivation of regorafenib-glucuronide in the gastrointestinal tract by *β*-glucuronidases is the major cause of gastrointestinal toxicity 164. Similar to that of CPT-11 and diclofenac, inhibition of microbial *β*-glucuronidases is believed to reduce regorafenib-induced toxicity 164. These studies suggested the possibility of the combinational use of bacterial *β*-glucuronidases for reducing the toxicity of drugs; however, additional clinical studies should be carried out to prove the feasibility of this approach.

In addition to bacterial enzyme inhibitors, antibiotics and ethnomedicines are good alternatives for use in combinations as well. In the food safety field, animal studies have demonstrated that the combination of melamine and broad-spectrum antibiotics can significantly reduce kidney injury caused by melamine 165, and the mechanism is associated with the reduced transformation of melamine into cyanuric acid by gut microbiota 166,167. The combined use of chemical drugs and ethnomedicines is an emerging approach in recent years. Combination of *Flos lonicera* with metformin and combination of *Houttuynia cordata* with metformin have shown great potential to improve the efficacy in rats 168,169. However, the specific mechanisms of the combinational use of ethnomedicines and chemical drugs remain unknown and need to be explored further.

### Conventional modification of gut microbiota

Although antibiotics are widely used to modify the composition and function of gut microbiota, we will mainly discuss non-drug approaches that are applied to modify gut microbiota (Figure 7B). FMT is an approach that transfers the fecal microbiota from the healthy donors to the gut of patients with perturbed microbiota. Clinical studies have shown that this method is a promising therapeutic option to treat diseases such as *Clostridioides difficile* infection and ulcerative colitis 170,171, although the feasibility, efficacy and safety of this approach need to be studied further 172,173. Currently, this approach is subject to many obstacles that can lead to variability of the samples received by recipients. One typical challenge of this approach is the quality of donor samples, since the stool samples are obtained from different donors, and the samples collected from the same donor can vary greatly at different time points. Fortunately, improvements in donor screening, preparation of fecal samples, sample banking, fecal delivery, clinical management, and the requirements for implementation of an FMT center have been made to overcome these obstacles 174-176. These advances in the standardization of FMT enable not only the direct treatment of diseases such as *Clostridioides difficile* infection and ulcerative colitis but also modification of drug efficacy and toxicity. Immune checkpoint inhibitors usually cause severe toxic events such as diarrhea and colitis 177. A recent clinical study showed that FMT can rapidly and significantly ameliorate colitis caused by immune checkpoint inhibitors 178, exemplifying the use of FMT to reduce the toxicity of drugs. FOLFOX, a regimen consisting of 5-FU, leucovorin, and oxaliplatin, is extensively applied for the treatment of colorectal cancer. However, this chemotherapy usually leads to intestinal mucositis. In another study, FMT significantly decreased diarrhea and intestinal mucositis caused by FOLFOX in a mouse model of colorectal cancer 179. Although the relevant cases are small in number and lack adequate evidence, these scattered cases point to the use of FMT as a promising method to manipulate the toxicity and efficacy of drugs.

Dietary intervention represents a good alternative to manipulate the composition and function of gut microbiota because of the convenience of this approach. It has been shown that the diet can rapidly and profoundly change gut microbiota and can also influence the interindividual variation of host microbial communities 180. In addition, nutritional compounds can be metabolized or transformed by gut microbiota into functional molecules with the ability to promote/inhibit the progression of diseases and interfere with the hepatic transformation of drugs, as in the case of *p*-cresol production 130. Thus, dietary intervention can be adopted to achieve the aim of precision medicine in the modification of drug efficacy and toxicity 181. A typical example of this approach is the use of the cardiac drug digoxin, whose toxicity is linked to the abundance of *E. lenta* carrying the *cgr* operon 126. In the study, the *in vitro* expression and activity of the *cgr* operon in *E. lenta* could be prevented by the amino acid arginine. Moreover, in gnotobiotic mice, the animals receiving a high-protein diet (high-arginine diet) and colonized with *cgr*-expressing *E. lenta* showed significantly higher concentrations of serum and urinary digoxin than the control mice. In contrast, the serum and urinary digoxin-elevating effects were not observed in mice colonized with *E. lenta* that lacked *cgr* operon. In another study, 3,4-dihydroxy- phenylacetic acid, a compound produced by gut microbiota metabolism of quercetin, attenuated acetaminophen-induced liver injury in mice 182. Similarly, urolithin A, a metabolite transformed from ellagic acid by gut microbiota, enhanced the therapeutic effects of 5-FU on cancer cells 183. These studies highlight the potential of using personalized dietary interventions to reduce the toxicity of drugs.

Prebiotics and probiotics have been extensively studied in the clinic for their beneficial effects on gut microbiota. Dietary supplementation with prebiotics promotes the growth of beneficial bacteria and the production of beneficial metabolites. For example, dietary supplementation with oligosaccharides promotes the growth of *Lactobacillus* and *Bifidobacterium* while inhibiting *Odoribacter* growth and increasing the fecal level of SCFAs in mice with constipation 184. Likewise, supplementation with probiotics such as *Bacillus*, *Escherichia*, and *Propionibacterium* can produce helpful effects on hosts by inhibiting the growth of harmful bacteria, producing beneficial metabolites, competing for nutrients, and facilitating the host immune response 185,186. For example, *Lactobacillus rhamnosus* synthesizes various molecules such as adhesive pili and lipoteichoic acid, and thus exerts immunomodulatory, probiotic, and antimicrobial effects 187. Because of the beneficial effects of prebiotics and probiotics, they are applied to correct the dysbiosis caused by diseases and drugs and to reduce the toxicity of drugs. One typical example is that the preventive administration of the *Lactobacillus casei* variety *rhamnosus* mitigated FOLFOX-induced diarrhea and intestinal mucositis in a mouse model of colorectal cancer 188. Similarly, combined administration of TGF-*β* blockade and *Escherichia coli* Nissle 1917, a probiotic strain, significantly improved the antitumor and immune suppression effects of TGF-*β* blockade in mice 189. However, the reliability of supplementing prebiotics and probiotics remains to be questioned because of the lack of strong clinical evidence and the negative experimental results. For example, one study showed that prebiotics, including fructo-, galacto-, and mannan-oligosaccharide, cannot protect rats from 5-FU-induced intestinal mucositis 190.

### Emerging engineering of gut microbiota

Although conventional approaches for the modification of gut microbiota have shown great potential for precision medicine, these approaches usually lack specificity as they may lead to extensive modification of untargeted gut bacteria. For example, the antibiotics prescribed for the elimination of specific bacteria can produce extensive and long-lasting effects on other gut bacteria and even influence the metabolism of hosts 191. The use of probiotics is more highly specific than other conventional approaches, such as the use of antibiotics and FMT; however, the functions of probiotics are limited because of the limited numbers of probiotics that have been discovered. With the rapid growth of synthetic biology, cellular behavior and function can be programmed with the use of natural and synthetic biological components. Advances in synthetic biology have tremendously improved the utilization of organisms for purposes ranging from the biosynthesis of chemical products to the development of complex therapeutics 192. In recent years, researchers have used synthetic biology to examine gut microbiota and hence have contributed to the development of microbiome engineering 193. Gut bacteria can be engineered for precision medicine by conferring bacteria with the ability to report surrounding signals, by precise deletion of bacterial strains, by endowing bacteria with the capability to release therapeutic molecules, and by restricting the proliferation of engineered bacteria (Figure 8).

Living organisms in different kingdoms have evolved to the use of biosensors to detect signals from the surrounding environment, such as temperature, pH, nutrients and the existence of other competitors through a series of complicated communications between and within species. With these biosensors, a cascade of cellular responses can be elicited by environmental stress and chemical stimuli. The genes encoding these sensors can be ported into other gut bacteria to report the surrounding signals. One typical example was shown in the study conducted by Daeffler *et al.* In their study, they engineered gut bacteria to have the capability to sense and report the levels of two compounds in mice with dextran sodium sulfate-induced colitis 194. Through a bioinformatics-based approach, two sensors including an improved sensor for tetrathionate and a novel sensor for thiosulfate were identified in the marine bacteria *Salmonella typhimurium* and *Shewanella baltica*. The two sensors were reprogrammed to drive the expression of green fluorescent protein and were ported to the gut‐adapted probiotic *E. coli* Nissle 1917. After the gavage of engineered strains into mice, flow cytometry was used to detect the engineered bacteria in the colon and feces and to analyze the sensor outputs. The results showed that the engineered thiosulfate sensor was activated by colon inflammation, suggesting the success of the biosensor in detecting colon inflammation and the thiosulfate content. In addition to tetrathionate and thiosulfate, gut bacteria have been engineered to sense and report signals that include but are not limited to cytokines such as TNF, IFN-*γ* and IL-1*β*, hormones such as adrenaline, metabolites such as γ-aminobutyric acid, physiological stimuli such as temperature, and quorum sensing signals (signals that carry the density information for the local population of a bacterial species or other bacterial species and guide the density-dependent bacterial responses) (Figure 8A).

Because the signals sensed and reported by engineered bacteria are so comprehensive that they almost encompass all the signals from surrounding bacteria, an emerging aim of bacteria engineering is the development of gut bacteria as diagnostic tools 193,195. As mentioned above, gut bacteria, enzymes, genes and metabolites can serve as biomarkers for precision medicine, yet not all of these biomarkers can be screened by conventional approaches because some of the biomarkers are easy to be degraded and hence are not easy to be detected. For example, the detection of metabolites usually requires a complex process of sample separation and extraction from hosts, and many metabolites can be degraded or transformed during this process. In contrast, the engineered diagnostic bacteria can function either directly within the body or indirectly in *ex vivo* clinical samples 196,197. Thus, compared with traditional methods, the use of engineered bacteria possesses the advantage of capturing transient molecules that can be easily degraded, absorbed or modified by gut microbiota or hosts 198. Correspondingly, engineered bacteria can be used as surrogate biomarkers for signals that cannot be detected by conventional methods and thus enable patient stratification and treatment decision when using drugs (Figure 8B). Another advantage of engineered diagnostic bacteria is that they can be equipped with other functions, such as the synthesis and release of therapeutic molecules with a variety of capabilities for modulating the host immune system and altering drug metabolizing ability.

For some drugs, interindividual differences in efficacy and toxicity can be ascribed only to certain gut bacterial species; hence, what we desire is the precise depletion of the species without significant modification of the levels of other bacteria. One attractive approach for achieving this goal is to utilize phages to selectively delete those culprits in the gut. Phages are naturally present in gut microbiota and are important for maintaining the balance of gut microbiota 199. One opinion is that phages have developed a balanced relationship with gut bacteria, as manifested by their co-existence in nature, making the depletion of specific gut bacteria impossible. However, evidence has supported the utilization of phages to modulate the gut microbiota composition 200,201. There are two approaches to using phages: one is to use phages in their pristine form by fecal filtrate transfer, a method that has been successfully used to treat patients with *C. difficile* infection (although in this case the metabolites may also contribute to the efficacy of treatment) 202, and the other is to confer stronger and more specific bacteria-killing effects by engineering the phages. For example, with the help of the clustered regularly interspaced short palindromic repeat (CRISPR)-Cas9 system, RNA-guided nucleases targeting specific DNA sequences can be efficiently delivered to microbial populations by phages 203. Using this strategy, Bikard *et al.* showed that reprogramming of Cas9 to target virulent genes can efficiently kill the virulent* Staphylococcus aureus*, but not the avirulent strain in mice 204. The targets of this strategy can be undesirable genes or polymorphisms that are associated with antibiotic resistance and virulence and are expected to be expanded to genes encoding drug metabolism enzymes. Taken together, the evidence shows that phages, especially engineered phages, are promising tools for precise depletion of gut bacteria (Figure 8C).

In contrast to the precise removal of gut bacteria, introducing engineered bacteria into gut, a method similar to the use of probiotics, can also confer beneficial effects to hosts (Figure 8D). Unlike the natural probiotics that only have the functions obtained during the process of natural evolution, engineered strains can be bestowed with genes of special interest to allow them to perform functions that are absent from hosts. Phenylketonuria is a genetic disease that is caused defects in the gene encoding phenylalanine hydroxylase, an enzyme that can metabolize the amino acid phenylalanine. Isabella* et al.* conferred *E. coli* Nissle with the ability to express genes encoding phenylalanine-metabolizing enzymes 205. Administration of SYNB1618, the engineered strain of* E. coli*, into phenylketonuria model mice reduced the blood concentration of phenylalanine by 38% in comparison with that in the control mice, and the reduction was independent of dietary protein intake. Another advantage of this study was that, hippurate, the final product of phenylalanine excreted in the urine, can act as a predictive biomarker for strain activity. In addition to phenylketonuria, bacteria have been engineered for treatment of cholera, colitis, type 1 diabetes, *Pseudomonas aeruginosa* infections, obesity, *etc*., by conferring bacteria with the capability to produce proteins, polypeptides and metabolites exhibiting immunoregulation and blood glucose regulation abilities such as IL-10, CAI-1, bacteriocin and linoleic acid isomerase 193. The success of these studies in animals has greatly encouraged and inspired researchers to prove the feasibility of this approach in the clinic.

To survive and thrive, the introduced strains have to compete with native gut bacteria that share similar niches. Once the goal of introducing engineered strains into the gut is achieved, the scenario we don't want to see is that the introduced strains profoundly perturbed the original gut microbiota composition, or overexpression of targeted genes. Hence, the ideal introduced strains are those that carry a “switch” that can be used to precisely control the proliferation of strains. Or in an alternative way, their expression of targeted genes can be regulated. To control the proliferation of specific strains, the most commonly used strategy is to engineer the strains to have auxotrophy (Figure 8E). An example of this strategy is the engineered strain SYNB1020, whose proliferation within and outside the host in the absence of thymidine is impaired by removal of the thymidylate synthase gene *thy*A 206. In a similar study, deletion of the *dap*A gene, encoding 4-hydroxytetrahydropicolinate synthase, enables biocontainment by rendering the cell wall biosynthesis and growth of the engineered strain SYNB1618 dependent on exogenous diaminopimelate 205. These two examples illustrate the strategy of using auxotrophy to control the proliferation of engineered strains. In addition to proliferation control, another approach to guarantee the normal function of other gut bacteria is to control the expression of genes using methods such as CRISPR interference (CRISPRi). CRISPRi utilizes dCas9, a mutant form of Cas9 that can bind but not cleave DNA, to regulate gene expression. This method has been used to induce sequence-specific, targeted knockdown of genes in gut bacteria 207.

Currently, microbiome engineering is an exciting and promising approach that can be used for diagnostic and therapeutic applications 198. Here we discussed major strategies that can be used for engineering the microbiome. With the development of new genetic tools and complex genetic circuits, synthetic biology will support more precise compositional and functional manipulation of gut microbiota. These genetic tools will enable further progress in the precision control of drug outcomes.

## Considerations and perspectives

In clinical practice, attention should be paid to the monitoring of biomarkers and the methods of manipulating gut microbiota. The function of gut microbiota and other factors that can shape drug outcomes should be monitored not only before the usage of drugs but also during the drug treatment. This is because the composition and function of gut microbiota are rather dynamic and are highly influenced by many factors such as emotional stress, diet, and exercise (Figure 4). In addition, the methods that aim to improve drug outcomes themselves can also impact the gut microbiota and the status of hosts. As a result, the levels of biomarkers are rather dynamic during the process of treatment, and the methods or dosage used for treatment should be adjusted according to the levels of the biomarkers 208. The combination of gut microbiota-oriented methods can be applied to improve drug outcomes. In Section 6, we discussed the methods used to improve drug outcomes, and these methods are usually applied individually. In clinical practice, an emerging frontier in gastrointestinal medicine is combinatory utilization of such methods to modulate the gut microbiota. FMT is highly efficacious for treatment of *Clostridium difficile* infection; however, single FMT is still not effective in about 10-20% of patients. Treatment with antibiotics can increase the colonization of implanted bacteria, and thus enhance the efficacy of FMT in rats 209. Prebiotic treatment combined with probiotic treatment allows more effective growth of probiotics in a gastrointestinal tract simulator 210. Therefore, we believe that the combined use of methods targeting gut microbiota can improve drug outcomes, and additional studies are needed to validate this hypothesis.

Although great progress in the standardization of donor screening, sample preparation, banking and delivery have been made concerning conventional methods such as FMT 174-176, new policies and regulatory frameworks are needed to evaluate the efficacy and safety of emerging approaches and the scientific validity of implementation protocols. Because most of the emerging approaches are performed mainly in animals, the safety and efficacy of these approaches in humans need to be strictly studied. Notably, since horizontal gene transfer is very common in the human gut microbiota, genetically modified DNA can spread from engineered bacteria to other endogenous members of gut microbiota 211. To make the matters worse, engineered bacteria can be released into the environment and further colonize other humans and animals, especially animals that are intolerant of such strains, raising the serious possibility of gene contamination. Although genetically modified strains seem to be less fit than wild strains, and kill switches have been developed to guarantee biocontainment 212,213, there is still the possibility of their spread to other humans and animals, and thus, the utilization of engineered bacteria should be carefully examined.

Direct mining of drugs from the gut is a promising approach that can minimize the variability of drug responses induced by gut microbiota. The gut microbiota can synthesize numerous natural products that enable microbe-microbe communication and microbial modulation of the host immune, nervous and metabolomic systems. However, the identities and functions of these molecules, for the most part, remain unknown. Currently, a burgeoning field pertaining to gut microbiota is to mine molecules with powerful bioactivities, and this field has generated plenty exciting achievements 214,215. These molecules are expected to be exploited as novel antibiotics, immunoregulators, anti-inflammatory and anti-obesity drugs, *etc.* In contrast to drugs mined outside of the gut that can undergo metabolism by gut microbiota, drugs mined from the gut may bypass the metabolic effects of gut microbiota and thus eliminate the variability of the drug response induced by gut microbiota.

There are two types of drugs that can be mined in the gut: molecules that are synthesized *de novo* by gut microbiota and molecules that are transformed from orally administered drugs. In addition to avoiding metabolism by gut microbiota, the drugs mined by this approach possess several other advantages. For example, traditional antibiotics often cause long-term and widespread destruction of the gut microbiota composition and even lead to antibiotic resistance, which may further promote the development and aggravation of diseases 216. In contrast, the antimicrobial molecules obtained from gut microbiota such as bacteriocins are outstanding candidates that can be developed as novel antibiotics since they can selectively eliminate particular strains 214,217. The high toxicity and poor bioavailability and efficacy of compounds in ethnomedicines often prevent the use of many ethnomedicines in the clinic. Compounds in ethnomedicines transformed by gut microbiota can be developed as new drugs as long as they show reduced toxicity and increased bioavailability and efficacy in comparison with the parent compounds.

## Abbreviations

5-FU: 5-fluorouracil
BCAAs: branched-chain amino acids
CTLA-4: cytotoxic T-lymphocyte-associated antigen-4
BVU: (*E*)-5-(2-bromovinyl) uracil
CRISPR: clustered regularly interspaced short palindromic repeat
FMT: fecal microbiota transplantation
GLP-1: glucagon-like peptide 1
HMG-CoA: *β*-hydroxy-*β*-methylglutaryl Coenzyme A
MS: mass spectrometry
NMR: nuclear magnetic resonance
NSAIDs: nonsteroidal anti-inflammatory drug
PD-1: programmed cell death protein-1
PP: 1-phenyl-1,2-propanedione
SCFAs: short-chain fatty acids
TMAO: trimethylamine N-oxide
TMA: trimethylamine
SULT1A1: sulfotransferase 1A1

## References

[1] Wilkinson GR (2005). Drug metabolism and variability among patients in drug response. N Engl J Med.

[2] Spear BB, Heath-Chiozzi M, Huff J (2001). Clinical application of pharmacogenetics. Trends Mol Med.

[3] Budnitz DS, Lovegrove MC, Crosby AE (2011). Emergency department visits for overdoses of acetaminophen-containing products. Am J Prev Med.

[4] Blieden M, Paramore LC, Shah D, Ben-Joseph R (2014). A perspective on the epidemiology of acetaminophen exposure and toxicity in the United States. Expert Rev Clin Pharmacol.

[5] Hisada T, Endoh K, Kuriki K (2015). Inter-and intra-individual variations in seasonal and daily stabilities of the human gut microbiota in Japanese. Arch Microbiol.

[6] Qin J, Li R, Raes J, Arumugam M, Burgdorf KS, Manichanh C (2010). A human gut microbial gene catalogue established by metagenomic sequencing. Nature.

[7] Scheline RR (1973). Metabolism of foreign compounds by gastrointestinal microorganisms. Pharmacol Rev.

[8] Carmody RN, Turnbaugh PJ (2014). Host-microbial interactions in the metabolism of therapeutic and diet-derived xenobiotics. J Clin Invest.

[9] Koppel N, Rekda VM, Balskus EP (2017). Chemical transformation of xenobiotics by the human gut microbiota. Science.

[10] Clarke G, Sandhu KV, Griffin BT, Dinan TG, Cryan JF, Hyland NP (2019). Gut reactions: breaking down xenobiotic-microbiome interactions. Pharmacol Rev.

[11] Amedei A, Boem F (2018). I've gut A feeling: microbiota impacting the conceptual and experimental perspectives of personalized medicine. Int J Mol Sci.

[12] Petrosino JF (2018). The microbiome in precision medicine: the way forward. Genome Med.

[13] Behrouzi A, Nafari AH, Siadat SD (2019). The significance of microbiome in personalized medicine. Clin Trans Med.

[14] Kashyap PC, Chia N, Nelson H, Segal E, Elinav E (2017). Microbiome at the frontier of personalized medicine. Mayo Clin Proc.

[15] Kuntz TM, Gilbert JA (2017). Introducing the microbiome into precision medicine. Trends Pharmacol Sci.

[16] Zmora N, Zeevi D, Korem T, Segal E, Elinav E (2016). Taking it personally: personalized utilization of the human microbiome in health and disease. Cell Host Microbe.

[17] Lam KN, Alexander M, Turnbaugh PJ (2019). Precision medicine goes microscopic: engineering the microbiome to improve drug outcomes. Cell Host Microbe.

[18] Sousa T, Paterson R, Moore V, Carlsson A, Abrahamsson B, Basit AW (2008). The gastrointestinal microbiota as a site for the biotransformation of drugs. Int J Pharm.

[19] Xu J, Chen HB, Li SL (2017). Understanding the molecular mechanisms of the interplay between herbal medicines and gut microbiota. Med Res Rev.

[20] McHan F, Shotts EB (1993). Effect of short-chain fatty acids on the growth of Salmonella typhimurium in an *in vitro* system. Avian Dis.

[21] Cardona F, Andrés-Lacueva C, Tulipani S, Tinahones FJ, Queipo-Ortuño MI (2013). Benefits of polyphenols on gut microbiota and implications in human health. J Nutr Biochem.

[22] Bazzocco S, Mattila I, Guyot S, Renard CM, Aura AM (2008). Factors affecting the conversion of apple polyphenols to phenolic acids and fruit matrix to short-chain fatty acids by human faecal microbiota *in vitro*. Eur J Nutr.

[23] Roager HM, Hansen LB, Bahl MI, Frandsen HL, Carvalho V, Gøbel RJ (2016). Colonic transit time is related to bacterial metabolism and mucosal turnover in the gut. Nat Microbiol.

[24] Müller M, Canfora EE, Blaak EE (2018). Gastrointestinal transit time, glucose homeostasis and metabolic health: modulation by dietary fibers. Nutrients.

[25] Zhou Y, Liu Z, Chen Y, Jin LH (2016). Identification of the protective effects of traditional medicinal plants against SDS-induced Drosophila gut damage. Exp Ther Med.

[26] Feng W, Ao H, Peng C, Yan D (2019). Gut microbiota, a new frontier to understand traditional Chinese medicines. Pharmacol Res.

[27] Reuter BK, Davies NM, Wallace JL (1997). Nonsteroidal anti-inflammatory drug enteropathy in rats: role of permeability, bacteria, and enterohepatic circulation. Gastroenterology.

[28] Nicholson JK, Holmes E, Kinross J, Burcelin R, Gibson G, Jia W (2012). Host-gut microbiota metabolic interactions. Science.

[29] Kalantar-Zadeh K, Berean KJ, Burgell RE, Muir JG, Gibson PR (2019). Intestinal gases: influence on gut disorders and the role of dietary manipulations. Nat Rev Gastroenterol Hepatol.

[30] Feng W, Ao H, Peng C (2018). Gut microbiota, short-chain fatty acids, and herbal medicines. Front Pharmacol.

[31] Kaddurah-Daouk R, Baillie RA, Zhu H, Zeng ZB, Wiest MM, Nguyen UT (2011). Enteric microbiome metabolites correlate with response to simvastatin treatment. PLoS One.

[32] Velasquez MT, Ramezani A, Manal A, Raj DS (2016). Trimethylamine N-oxide: the good, the bad and the unknown. Toxins.

[33] Niehues M, Hensel A (2009). In-vitro interaction of L-dopa with bacterial adhesins of Helicobacter pylori: an explanation for clinicial differences in bioavailability?. J Pharm Pharmacol.

[34] Slyepchenko A, Maes M, Jacka FN, Köhler CA, Barichello T, McIntyre RS (2017). Gut microbiota, bacterial translocation, and interactions with diet: pathophysiological links between major depressive disorder and non-communicable medical comorbidities. Psychother Psychosom.

[35] Vrakas S, Mountzouris KC, Michalopoulos G, Karamanolis G, Papatheodoridis G, Tzathas C (2017). Intestinal bacteria composition and translocation of bacteria in inflammatory bowel disease. PLoS One.

[36] Giannelli V, Di Gregorio V, Iebba V, Giusto M, Schippa S, Merli M (2014). Microbiota and the gut-liver axis: bacterial translocation, inflammation and infection in cirrhosis. World J Gastroenterol.

[37] Tinkov AA, Gritsenko VA, Skalnaya MG, Cherkasov SV, Aaseth J, Skalny AV (2018). Gut as a target for cadmium toxicity. Environ Pollut.

[38] Lindenbaum J, Rund DG, Butler Jr VP, Tse-Eng D, Saha JR (1981). Inactivation of digoxin by the gut flora: reversal by antibiotic therapy. N Engl J Med.

[39] Peters U, Falk LC (1978). Kalman SM. Digoxin metabolism in patients. Arch Intern Med.

[40] Peppercorn MA, Goldman P (1972). The role of intestinal bacteria in the metabolism of salicylazosulfapyridine. J Pharm Exp Ther.

[41] Guo C, Liu S, Guo Y, Yin Y, Lin J, Chen X (2014). Comparative function-structural analysis of antiplatelet and antiradical activities of flavonoid phytochemicals. J Anim Plant Sci.

[42] Yang B, Liu H, Yang J, Gupta VK, Jiang Y (2018). New insights on bioactivities and biosynthesis of flavonoid glycosides. Trends Food Sci Technol.

[43] Wright B, Spencer JP, Lovegrove JA, Gibbins JM (2013). Insights into dietary flavonoids as molecular templates for the design of anti-platelet drugs. Cardiovasc Res.

[44] Laparra JM, Sanz Y (2010). Interactions of gut microbiota with functional food components and nutraceuticals. Pharmacol Res.

[45] Wu TR, Lin CS, Chang CJ, Lin TL, Martel J, Ko YF (2019). Gut commensal Parabacteroides goldsteinii plays a predominant role in the anti-obesity effects of polysaccharides isolated from Hirsutella sinensis. Gut.

[46] Yue SJ, Liu J, Wang AT, Meng XT, Yang ZR, Peng C (2019). Berberine alleviates insulin resistance by reducing peripheral branched-chain amino acids. Am J Physiol Endocrinol Metab.

[47] Dunn CJ, Peters DH (1995). Metformin. Drugs.

[48] Buse JB, DeFronzo RA, Rosenstock J, Kim T, Burns C, Skare S (2016). The primary glucose-lowering effect of metformin resides in the gut, not the circulation: results from short-term pharmacokinetic and 12-week dose-ranging studies. Diabetes Care.

[49] Pryor R, Martinez-Martinez D, Quintaneiro L, Cabreiro F (2020). The role of the microbiome in drug response. Annu Rev Pharmacol Toxicol.

[50] Viaud S, Saccheri F, Mignot G, Yamazaki T, Daillère R, Hannani D (2013). The intestinal microbiota modulates the anticancer immune effects of cyclophosphamide. Science.

[51] Reiner Ž (2014). Resistance and intolerance to statins. Nutr Metab Cardiovasc Dis.

[52] Mangravite LM, Thorn CF, Krauss RM (2006). Clinical implications of pharmacogenomics of statin treatment. Pharmacogenomics J.

[53] Dawson PA, Lan T, Rao A (2009). Bile acid transporters. J Lipid Res.

[54] Wang L, Wang Y, Wang H, Zhou X, Wei X, Xie Z (2018). The influence of the intestinal microflora to the efficacy of Rosuvastatin. Lipids Health Dis.

[55] Caparrós-Martín JA, Lareu RR, Ramsay JP, Peplies J, Reen FJ, Headlam HA (2017). Statin therapy causes gut dysbiosis in mice through a PXR-dependent mechanism. Microbiome.

[56] Vanhoefer U, Harstrick A, Achterrath W, Cao S, Seeber S, Rustum YM (2001). Irinotecan in the treatment of colorectal cancer: clinical overview. J Clin Oncol.

[57] Wiseman LR (1996). Markham A. Irinotecan. Drugs.

[58] Saitta KS, Zhang C, Lee KK, Fujimoto K, Redinbo MR, Boelsterli UA (2014). Bacterial β-glucuronidase inhibition protects mice against enteropathy induced by indomethacin, ketoprofen or diclofenac: mode of action and pharmacokinetics. Xenobiotica.

[59] Volkova M, Russell R (2011). Anthracycline cardiotoxicity: prevalence, pathogenesis and treatment. Curr Cardiol Rev.

[60] Yan A, Culp E, Perry J, Lau JT, MacNeil LT, Surette MG (2018). Transformation of the anticancer drug doxorubicin in the human gut microbiome. ACS Infect Dis.

[61] Kang MJ, Khanal T, Kim HG, Lee DH, Yeo HK, Lee YS (2012). Role of metabolism by human intestinal microflora in geniposide-induced toxicity in HepG2 cells. Arch Pharm Res.

[62] Zhang M, Peng C, Li X (2015). *In vivo* and *in vitro* metabolites from the main diester and monoester diterpenoid alkaloids in a traditional chinese herb, the aconitum species. Evid Based Complement Alternat Med. 2015.

[63] Wang S, Wu X, Tan M, Gong J, Tan W, Bian B (2012). Fighting fire with fire: poisonous Chinese herbal medicine for cancer therapy. J Ethnopharmacol.

[64] Efferth T, Li PC, Konkimalla VSB, Kaina B (2007). From traditional Chinese medicine to rational cancer therapy. Trends Mol Med.

[65] Hinrichsen S, Geist F, Planer-Friedrich B (2015). Inorganic and methylated thioarsenates pass the gastrointestinal barrier. Chem Res Toxicol.

[66] Watanabe T, Hirano S (2013). Metabolism of arsenic and its toxicological relevance. Arch Toxicol.

[67] Koontz JM, Dancy BC, Horton CL, Stallings JD, DiVito VT, Lewis JA (2019). The role of the human microbiome in chemical toxicity. Int J Toxicol.

[68] Zhou B, Xia X, Wang P, Chen S, Yu C, Huang R (2018). Induction and amelioration of methotrexate-induced gastrointestinal toxicity are related to immune response and gut microbiota. EBioMedicine.

[69] Assefa S, Köhler G (2020). Intestinal microbiome and metal toxicity. Curr Opin Toxicol.

[70] Lu K, Abo RP, Schlieper KA, Graffam ME, Levine S, Wishnok JS (2014). Arsenic exposure perturbs the gut microbiome and its metabolic profile in mice: an integrated metagenomics and metabolomics analysis. Environ Health Perspect.

[71] Choiniere J, Wang L (2016). Exposure to inorganic arsenic can lead to gut microbe perturbations and hepatocellular carcinoma. Acta Pharm Sin B.

[72] Chiocchetti GM, Vélez D, Devesa V (2018). Effect of subchronic exposure to inorganic arsenic on the structure and function of the intestinal epithelium. Toxicol Lett.

[73] Hodgman MJ (2012). Garrard AR. A review of acetaminophen poisoning. Crit Care Clin.

[74] Duan SX, von Moltke LL, Greenblatt DJ, Patten CJ, Miners JO, Mackenzie PI (2001). Interindividual variability in acetaminophen glucuronidation by human liver microsomes: identification of relevant acetaminophen UDP-glucuronosyltransferase isoforms. J Pharmacol Exp Ther.

[75] Laine JE, Auriola S, Pasanen M, Juvonen RO (2009). Acetaminophen bioactivation by human cytochome P450 enzymes and animal microsomes. Xenobiotica.

[76] Gamage N, Barnett A, Hempel N, Duggleby RG, Windmill KF, Martin JL (2006). Human sulfotransferases and their role in chemical metabolism. Toxicol Sci.

[77] Clayton TA, Baker D, Lindon JC, Everett JR, Nicholson JK (2009). Pharmacometabonomic identification of a significant host-microbiome metabolic interaction affecting human drug metabolism. Proc Natl Acad Sci U S A.

[78] Brochado AR, Telzerow A, Bobonis J, Banzhaf M, Mateus A, Selkrig J (2018). Species-specific activity of antibacterial drug combinations. Nature.

[79] Nakayama H, Kinouchi T, Kataoka K, Akimoto S, Matsuda Y, Ohnishi Y (1997). Intestinal anaerobic bacteria hydrolyse sorivudine, producing the high blood concentration of 5-(E)-(2-bromovinyl) uracil that increases the level and toxicity of 5-fluorouracil. Pharmacogenetics.

[80] Okuda H, Nishiyama T, Ogura K, Nagayama S, Ikeda K, Yamaguchi S (1997). Lethal drug interactions of sorivudine, a new antiviral drug, with oral 5-fluorouracil prodrugs. Drug Metab Dispos.

[81] Wang S, Hu Y, Tan W, Wu X, Chen R, Cao J (2012). Compatibility art of traditional Chinese medicine: from the perspective of herb pairs. J Ethnopharmacol.

[82] Li S, Zhang B (2013). Traditional Chinese medicine network pharmacology: theory, methodology and application. Chin J Nat Med.

[83] Wang M, Chen L, Liu D, Chen H, Tang DD, Zhao YY (2017). Metabolomics highlights pharmacological bioactivity and biochemical mechanism of traditional Chinese medicine. Chem Biol Interact.

[84] Zhou SS, Xu J, Zhu H, Wu J, Xu JD, Yan R (2016). Gut microbiota-involved mechanisms in enhancing systemic exposure of ginsenosides by coexisting polysaccharides in ginseng decoction. Sci Rep.

[85] Yue SJ, Wang WX, Chen YY, Shi XQ, Yan D, Yu JG (2019). Gut microbiota modulation with traditional Chinese medicine: a system biology-driven approach. Pharmacol. Res.

[86] Routy B, Le Chatelier E, Derosa L, Duong CP, Alou MT, Daillère R (2018). Gut microbiome influences efficacy of PD-1-based immunotherapy against epithelial tumors. Science.

[87] Zheng Y, Wang T, Tu X, Huang Y, Zhang H, Tan D (2019). Gut microbiome affects the response to anti-PD-1 immunotherapy in patients with hepatocellular carcinoma. J Immunother Cancer.

[88] Gopalakrishnan V, Spencer CN, Nezi L, Reuben A, Andrews MC, Karpinets TV (2018). Gut microbiome modulates response to anti-PD-1 immunotherapy in melanoma patients. Science.

[89] Alolga RN, Fan Y, Chen Z, Liu LW, Zhao YJ, Li J (2016). Significant pharmacokinetic differences of berberine are attributable to variations in gut microbiota between Africans and Chinese. Sci Rep.

[90] Li L, Ning Z, Zhang X, Mayne J, Cheng K, Stintzi A, Figeys D (2020). RapidAIM: A culture-and metaproteomics-based Rapid Assay of Individual Microbiome responses to drugs. Microbiome.

[91] Vila AV, Collij V, Sanna S, Sinha T, Imhann F, Bourgonje AR (2020). Impact of commonly used drugs on the composition and metabolic function of the gut microbiota. Nat Commun.

[92] Zimmermann M, Zimmermann-Kogadeeva M, Wegmann R, Goodman AL (2019). Mapping human microbiome drug metabolism by gut bacteria and their genes. Nature.

[93] Benson AK, Kelly SA, Legge R, Ma F, Low SJ, Kim J (2010). Individuality in gut microbiota composition is a complex polygenic trait shaped by multiple environmental and host genetic factors. Proc Natl Acad Sci U S A.

[94] Rothschild D, Weissbrod O, Barkan E, Kurilshikov A, Korem T, Zeevi D (2018). Environment dominates over host genetics in shaping human gut microbiota. Nature.

[95] Khachatryan ZA, Ktsoyan ZA, Manukyan GP, Kelly D, Ghazaryan KA, Aminov RI (2008). Predominant role of host genetics in controlling the composition of gut microbiota. PLoS One.

[96] Penders J, Thijs C, Vink C, Stelma FF, Snijders B, Kummeling I (2006). Factors influencing the composition of the intestinal microbiota in early infancy. Pediatrics.

[97] Quigley EM (2017). Gut microbiome as a clinical tool in gastrointestinal disease management: are we there yet?. Nat Rev Gastroenterol Hepatol.

[98] Huttenhower C, Gevers D, Knight R, Abubucker S, Badger JH, Chinwalla AT (2012). Structure, function and diversity of the healthy human microbiome. Nature.

[99] Lynch SV, Pedersen O (2016). The human intestinal microbiome in health and disease. N Engl J Med.

[100] Narhi LO, Fulco AJ (1982). Phenobarbital induction of a soluble cytochrome P-450-dependent fatty acid monooxygenase in Bacillus megaterium. J Biol Chem.

[101] Segata N, Haake SK, Mannon P, Lemon KP, Waldron L, Gevers D (2012). Composition of the adult digestive tract bacterial microbiome based on seven mouth surfaces, tonsils, throat and stool samples. Genome Biol.

[102] Franzosa EA, Morgan XC, Segata N, Waldron L, Reyes J, Earl AM (2014). Relating the metatranscriptome and metagenome of the human gut. Proc Natl Acad Sci U S A.

[103] Verberkmoes NC, Russell AL, Shah M, Godzik A, Rosenquist M, Halfvarson J (2009). Shotgun metaproteomics of the human distal gut microbiota. ISME J.

[104] Pollet RM, D'agostino EH, Walton WG, Xu Y, Little MS, Biernat KA (2017). An atlas of β-glucuronidases in the human intestinal microbiome. Structure.

[105] Wang Z, Klipfell E, Bennett BJ, Koeth R, Levison BS, DuGar B (2011). Gut flora metabolism of phosphatidylcholine promotes cardiovascular disease. Nature.

[106] Schwiertz A, Taras D, Schäfer K, Beijer S, Bos NA, Donus C, Hardt PD (2010). Microbiota and SCFA in lean and overweight healthy subjects. Obesity.

[107] Das A, Srinivasan M, Ghosh TS, Mande SS (2016). Xenobiotic metabolism and gut microbiomes. PLoS One.

[108] Chi L, Bian X, Gao B, Ru H, Tu P, Lu K (2016). Sex-specific effects of arsenic exposure on the trajectory and function of the gut microbiome. Chem Res Toxicol.

[109] Ozturk N, Ozturk D, Kavakli IH, Okyar A (2017). Molecular aspects of circadian pharmacology and relevance for cancer chronotherapy. Int J Mol Sci.

[110] Voigt RM, Forsyth CB, Green SJ (2016). Circadian rhythm and the gut microbiome. International review of neurobiology. Academic Press.

[111] Thaiss CA, Levy M, Korem T, Dohnalová L, Shapiro H, Jaitin DA (2016). Microbiota diurnal rhythmicity programs host transcriptome oscillations. Cell.

[112] Kim YC, Lee SJ (1998). Temporal variation in hepatotoxicity and metabolism of acetaminophen in mice. Toxicology.

[113] Kakan X, Chen P, Zhang J (2011). Clock gene mPer2 functions in diurnal variation of acetaminophen induced hepatotoxicity in mice. Exp Toxicol Pathol.

[114] Gong S, Lan T, Zeng L, Luo H, Yang X, Li N (2018). Gut microbiota mediates diurnal variation of acetaminophen induced acute liver injury in mice. J Hepatol.

[115] Zhao L (2013). The gut microbiota and obesity: from correlation to causality. Nat Rev Microbiol.

[116] Ni J, Wu GD, Albenberg L, Tomov VT (2017). Gut microbiota and IBD: causation or correlation?. Nat Rev Gastroenterol Hepatol.

[117] Rabbani G, Butler T, Knight J, Sanyal SC, Alam K (1987). Randomized controlled trial of berberine sulfate therapy for diarrhea due to enterotoxigenic Escherichia coli and Vibrio cholerae. J Infect Dis.

[118] Kong W, Wei J, Abidi P, Lin M, Inaba S, Li C (2004). Berberine is a novel cholesterol-lowering drug working through a unique mechanism distinct from statins. Nat Med.

[119] Yue SJ, Liu J, Wang WX, Wang AT, Yang XY, Guan HS (2019). Berberine treatment-emergent mild diarrhea associated with gut microbiota dysbiosis. Biomed Pharmacother.

[120] Lan J, Zhao Y, Dong F, Yan Z, Zheng W, Fan J, Sun G (2015). Meta-analysis of the effect and safety of berberine in the treatment of type 2 diabetes mellitus, hyperlipemia and hypertension. J Ethnopharmacol.

[121] Feng R, Shou JW, Zhao ZX, He CY, Ma C, Huang M (2015). Transforming berberine into its intestine-absorbable form by the gut microbiota. Sci Rep.

[122] Wang Y, Tong Q, Shou JW, Zhao ZX, Li XY, Zhang XF (2017). Gut microbiota-mediated personalized treatment of hyperlipidemia using berberine. Theranostics.

[123] Dobkin JF, Saha JR, Butler VP, Neu HC, Lindenbaum J (1983). Digoxin-inactivating bacteria: identification in human gut flora. Science.

[124] Mathan VI, Wiederman J, Dobkin JF, Lindenbaum J (1989). Geographic differences in digoxin inactivation, a metabolic activity of the human anaerobic gut flora. Gut.

[125] Koppel N, Bisanz JE, Pandelia ME, Turnbaugh PJ, Balskus EP (2018). Discovery and characterization of a prevalent human gut bacterial enzyme sufficient for the inactivation of a family of plant toxins. Elife.

[126] Haiser HJ, Gootenberg DB, Chatman K, Sirasani G, Balskus EP, Turnbaugh PJ (2013). Predicting and manipulating cardiac drug inactivation by the human gut bacterium Eggerthella lenta. Science.

[127] Venkataramanan R, Swaminathan A, Prasad T, Jain A, Zuckerman S, Warty V (1995). Clinical pharmacokinetics of tacrolimus. Clin Pharmacokinet.

[128] Lee JR, Muthukumar T, Dadhania D, Taur Y, Jenq RR, Toussaint NC (2015). Gut microbiota and tacrolimus dosing in kidney transplantation. PLoS One.

[129] Guo Y, Crnkovic CM, Won KJ, Yang X, Lee JR, Orjala J (2019). Commensal gut bacteria convert the immunosuppressant tacrolimus to less potent metabolites. Drug Metab Dispos.

[130] Spanogiannopoulos P, Bess EN, Carmody RN, Turnbaugh PJ (2016). The microbial pharmacists within us: a metagenomic view of xenobiotic metabolism. Nat Rev Microbiol.

[131] Matson V, Fessler J, Bao R, Chongsuwat T, Zha Y, Alegre ML (2018). The commensal microbiome is associated with anti-PD-1 efficacy in metastatic melanoma patients. Science.

[132] Weber JS, Dummer R, de Pril V, Lebbé C, Hodi FS (2013). Patterns of onset and resolution of immune-related adverse events of special interest with ipilimumab: detailed safety analysis from a phase 3 trial in patients with advanced melanoma. Cancer.

[133] Dubin K, Callahan MK, Ren B, Khanin R, Viale A, Ling L (2016). Intestinal microbiome analyses identify melanoma patients at risk for checkpoint-blockade-induced colitis. Nat Commun.

[134] Vétizou M, Pitt JM, Daillère R, Lepage P, Waldschmitt N, Flament C (2015). Anticancer immunotherapy by CTLA-4 blockade relies on the gut microbiota. Science.

[135] Chaput N, Lepage P, Coutzac C, Soularue E, Le Roux K, Monot C (2017). Baseline gut microbiota predicts clinical response and colitis in metastatic melanoma patients treated with ipilimumab. Ann Oncol.

[136] Siegel CA, Melmed GY (2009). Predicting response to anti-TNF agents for the treatment of Crohn's disease. Therap Adv Gastroenterol.

[137] Prasad V (2016). Perspective: The precision-oncology illusion. Nature.

[138] Innocenti F, Undevia SD, Chen PX, Das S, Ramirez J, Dolan ME (2004). Pharmacogenetic analysis of interindividual irinotecan (CPT-11) pharmacokinetic (PK) variability: evidence for a functional variant of ABCC2. J Clin Oncol.

[139] Lankisch TO, Schulz C, Zwingers T, Erichsen TJ, Manns MP, Heinemann V (2008). Gilbert's Syndrome and irinotecan toxicity: combination with UDP-glucuronosyltransferase 1A7 variants increases risk. Cancer Epidemiol Biomarkers Prev.

[140] Guthrie L, Gupta S, Daily J, Kelly L (2017). Human microbiome signatures of differential colorectal cancer drug metabolism. NPJ biofilms and microbiomes.

[141] Ong FS, Deignan JL, Kuo JZ, Bernstein KE, Rotter JI, Grody WW (2012). Clinical utility of pharmacogenetic biomarkers in cardiovascular therapeutics: a challenge for clinical implementation. Pharmacogenomics.

[142] Lurie Y, Loebstein R, Kurnik D, Almog S, Halkin H (2010). Warfarin and vitamin K intake in the era of pharmacogenetics. Br J Clin Pharmacol.

[143] Karl JP, Meydani M, Barnett JB, Vanegas SM, Barger K, Fu X (2017). Fecal concentrations of bacterially derived vitamin K forms are associated with gut microbiota composition but not plasma or fecal cytokine concentrations in healthy adults. Am J Clin Nutr.

[144] Violi F, Lip GY, Pignatelli P, Pastori D (2016). Interaction between dietary vitamin K intake and anticoagulation by vitamin K antagonists: is it really true?. Medicine.

[145] Zimmermann M, Zimmermann-Kogadeeva M, Wegmann R, Goodman AL (2019). Separating host and microbiome contributions to drug pharmacokinetics and toxicity. Science.

[146] Zimmermann-Kogadeeva M, Zimmermann M, Goodman A L (2019). Insights from pharmacokinetic models of host-microbiome drug metabolism. Gut Microbes.

[147] Arnold JW, Roach J, Azcarate-Peril MA (2016). Emerging technologies for gut microbiome research. Trends Microbiol.

[148] Whidbey C, Wright AT (2018). Activity-based protein profiling-enabling multimodal functional studies of microbial communities. In: Cravatt B, Hsu KL, Weerapana E. Current Topics in Microbiology and Immunology, Springer, Cham.

[149] Keller LJ, Babin BM, Lakemeyer M, Bogyo M (2020). Activity-based protein profiling in bacteria: Applications for identification of therapeutic targets and characterization of microbial communities. Curr Opin Chem Biol.

[150] Ananthakrishnan AN, Luo C, Yajnik V, Khalili H, Garber JJ, Stevens BW (2017). Gut microbiome function predicts response to anti-integrin biologic therapy in inflammatory bowel diseases. Cell Host Microbe.

[151] Noronha A, Modamio J, Jarosz Y, Guerard E, Sompairac N, Preciat G (2019). The Virtual Metabolic Human database: integrating human and gut microbiome metabolism with nutrition and disease. Nucleic Acids Res.

[152] Ford AC, Harris LA, Lacy BE, Quigley EM, Moayyedi P (2018). Systematic review with meta-analysis: the efficacy of prebiotics, probiotics, synbiotics and antibiotics in irritable bowel syndrome. Aliment Pharmacol Ther.

[153] Sanders ME, Merenstein DJ, Reid G, Gibson GR, Rastall RA (2019). Probiotics and prebiotics in intestinal health and disease: from biology to the clinic. Nat Rev Gastroenterol Hepatol.

[154] Tsai YL, Lin TL, Chang CJ, Wu TR, Lai WF, Lu CC (2019). Probiotics, prebiotics and amelioration of diseases. J Biomed Sci.

[155] Zhu W, Winter MG, Byndloss MX, Spiga L, Duerkop BA, Hughes ER (2018). Precision editing of the gut microbiota ameliorates colitis. Nature.

[156] Guthrie L, Kelly L (2019). Bringing microbiome-drug interaction research into the clinic. EBioMedicine.

[157] Chamseddine AN, Ducreux M, Armand JP, Paoletti X, Satar T (2019). Paci A, *et al*. Intestinal bacterial β-glucuronidase as a possible predictive biomarker of irinotecan-induced diarrhea severity. Pharmacol Ther.

[158] Pellock SJ, Creekmore BC, Walton WG, Mehta N, Biernat KA, Cesmat AP (2018). Gut microbial β-Glucuronidase inhibition via catalytic cycle interception. ACS Cent Sci.

[159] Achord DT, Brot FE, Bell CE, Sly WS (1978). Human β-glucuronidase: *in vivo* clearance and *in vitro* uptake by a glycoprotein recognition system on reticuloendothelial cells. Cell.

[160] Reddy BS, Weisburger JH, Wynder EL (1974). Fecal bacterial β-glucuronidase: control by diet. Science.

[161] Cheng KW, Tseng CH, Tzeng CC, Leu YL, Cheng TC, Wang JY (2019). Pharmacological inhibition of bacterial *β*-glucuronidase prevents irinotecan-induced diarrhea without impairing its antitumor efficacy *in vivo*. Pharmacol Res.

[162] Klein M, Andersen LPH, Harvald T, Rosenberg J, Gögenur I (2009). Increased risk of anastomotic leakage with diclofenac treatment after laparoscopic colorectal surgery. Dig Surg.

[163] Yauw ST, Arron M, Lomme RM, van den Broek P, Greupink R, Bhatt AP (2018). Microbial glucuronidase inhibition reduces severity of diclofenac-induced anastomotic leak in rats. Surg Infect.

[164] Ervin SM, Hanley RP, Lim L, Walton WG, Pearce KH, Bhatt AP (2019). Targeting regorafenib-induced toxicity through inhibition of gut microbial β-glucuronidases. ACS Chem Biol.

[165] Zheng X, Zhao A, Xie G, Chi Y, Zhao L, Li H (2013). Melamine-induced renal toxicity is mediated by the gut microbiota. Sci Transl Med.

[166] Jutzi K, Cook AM, Hütter R (1982). The degradative pathway of the s-triazine melamine. The steps to ring cleavage. Biochem J.

[167] Shelton DR, Karns JS, McCarty GW (1997). Durham DR. Metabolism of melamine by Klebsiella terragena. Appl Environ Microbiol.

[168] Shin NR, Bose S, Wang JH, Ansari A, Lim SK, Chin YW (2017). Flos lonicera combined with metformin ameliorates hepatosteatosis and glucose intolerance in association with gut microbiota modulation. Front Microbiol.

[169] Wang JH, Bose S, Lim SK, Ansari A, Chin YW, Choi HS (2017). Houttuynia cordata facilitates metformin on ameliorating insulin resistance associated with gut microbiota alteration in OLETF rats. Genes.

[170] Paramsothy S, Kamm MA, Kaakoush NO, Walsh AJ, van den Bogaerde J, Samuel D (2017). Multidonor intensive faecal microbiota transplantation for active ulcerative colitis: a randomised placebo-controlled trial. Lancet.

[171] Juul FE, Garborg K, Bretthauer M, Skudal H, Øines MN, Wiig H (2018). Fecal microbiota transplantation for primary Clostridium difficile infection. N Engl J Med.

[172] Li YT, Cai HF, Wang ZH, Xu J, Fang JY (2016). Systematic review with meta-analysis: long-term outcomes of faecal microbiota transplantation for Clostridium difficile infection. Aliment Pharmacol Ther.

[173] Costello SP, Soo W, Bryant RV, Jairath V, Hart AL, Andrews JM (2017). Systematic review with meta-analysis: faecal microbiota transplantation for the induction of remission for active ulcerative colitis. Aliment Pharmacol Ther.

[174] Ng SC, Kamm MA, Yeoh YK, Chan PK, Zuo T, Tang W (2020). Scientific frontiers in faecal microbiota transplantation: joint document of Asia-Pacific Association of Gastroenterology (APAGE) and Asia-Pacific Society for Digestive Endoscopy (APSDE). Gut.

[175] Cammarota G, Ianiro G, Kelly CR, Mullish BH, Allegretti JR, Kassam Z (2019). International consensus conference on stool banking for faecal microbiota transplantation in clinical practice. Gut.

[176] Kassam Z, Dubois N, Ramakrishna B, Ling K, Qazi T, Smith M (2019). Donor Screening for Fecal Microbiota Transplantation. N Engl J Med.

[177] Foppen MHG, Rozeman EA, van Wilpe S, Postma C, Snaebjornsson P, van Thienen JV (2018). Immune checkpoint inhibition-related colitis: symptoms, endoscopic features, histology and response to management. ESMO Open.

[178] Wang Y, Wiesnoski DH, Helmink BA, Gopalakrishnan V, Choi K, DuPont HL (2018). Fecal microbiota transplantation for refractory immune checkpoint inhibitor-associated colitis. Nat Med.

[179] Chang CW, Lee HC, Li LH, Chiang Chiau JS, Wang TE, Chuang WH (2020). Fecal microbiota transplantation prevents intestinal injury, upregulation of toll-like receptors, and 5-fluorouracil/oxaliplatin-induced toxicity in colorectal cancer. Int J Mol Sci.

[180] Carmody RN, Gerber GK, Luevano Jr JM, Gatti DM, Somes L, Svenson KL (2015). Diet dominates host genotype in shaping the murine gut microbiota. Cell host microbe.

[181] Sanderson SM, Gao X, Dai Z, Locasale JW (2019). Methionine metabolism in health and cancer: a nexus of diet and precision medicine. Nat. Rev. Cancer.

[182] Xue H, Xie W, Jiang Z, Wang M, Wang J, Zhao H (2016). 3, 4-Dihydroxyphenylacetic acid, a microbiota-derived metabolite of quercetin, attenuates acetaminophen (APAP)-induced liver injury through activation of Nrf-2. Xenobiotica.

[183] González-Sarrías A, Tomé-Carneiro J, Bellesia A, Tomás-Barberán FA, Espín JC (2015). The ellagic acid-derived gut microbiota metabolite, urolithin A, potentiates the anticancer effects of 5-fluorouracil chemotherapy on human colon cancer cells. Food Funct.

[184] Wang L, Hu L, Yan S, Jiang T, Fang S, Wang G (2017). Effects of different oligosaccharides at various dosages on the composition of gut microbiota and short-chain fatty acids in mice with constipation. Food Funct.

[185] Sánchez B, Delgado S, Blanco-Míguez A, Lourenço A, Gueimonde M, Margolles A (2017). Probiotics, gut microbiota, and their influence on host health and disease. Mol Nutr Food Res.

[186] McFarland LV (2014). Use of probiotics to correct dysbiosis of normal microbiota following disease or disruptive events: a systematic review. BMJ Open.

[187] Segers ME, Lebeer S (2014). Towards a better understanding of Lactobacillus rhamnosus GG-host interactions. Microb Cell Fact.

[188] Chang CW, Liu CY, Lee HC, Huang YH, Li LH, Chiau JSC (2018). Lactobacillus casei variety rhamnosus probiotic preventively attenuates 5-fluorouracil/oxaliplatin-induced intestinal injury in a syngeneic colorectal cancer model. Front Microbiol.

[189] Shi L, Sheng J, Wang M, Luo H, Zhu J, Zhang B (2019). Combination therapy of TGF-β blockade and commensal-derived probiotics provides enhanced antitumor immune response and tumor suppression. Theranostics.

[190] Yazbeck R, Lindsay RJ, Geier MS, Butler RN, Howarth GS (2019). Prebiotics fructo-, galacto-, and mannan-oligosaccharide do not protect against 5-fluorouracil-induced intestinal mucositis in rats. J Nutr.

[191] Pérez-Cobas AE, Gosalbes MJ, Friedrichs A, Knecht H, Artacho A, Eismann K (2013). Gut microbiota disturbance during antibiotic therapy: a multi-omic approach. Gut.

[192] Khalil AS, Collins JJ (2010). Synthetic biology: applications come of age. Nat Rev Genet.

[193] Inda ME, Broset E, Lu TK, de la Fuente-Nunez C (2019). Emerging frontiers in microbiome engineering. Trends Immunol.

[194] Daeffler KNM, Galley JD, Sheth RU, Ortiz-Velez LC, Bibb CO, Shroyer NF (2017). Engineering bacterial thiosulfate and tetrathionate sensors for detecting gut inflammation. Mol Syst Biol.

[195] Bober JR, Beisel CL, Nair NU (2018). Synthetic biology approaches to engineer probiotics and members of the human microbiota for biomedical applications. Annu Rev Biomed Eng.

[196] Riglar DT, Giessen TW, Baym M, Kerns SJ, Niederhuber MJ, Bronson RT (2017). Engineered bacteria can function in the mammalian gut long-term as live diagnostics of inflammation. Nat Biotechnol.

[197] Courbet A, Endy D, Renard E, Molina F, Bonnet J (2015). Detection of pathological biomarkers in human clinical samples via amplifying genetic switches and logic gates. Sci Transl Med.

[198] Riglar DT, Silver PA (2018). Engineering bacteria for diagnostic and therapeutic applications. Nat Revs Microbiol.

[199] Manrique P, Bolduc B, Walk ST, van der Oost J, de Vos WM, Young MJ (2016). Healthy human gut phageome. Proc Natl Acad Sci U S A.

[200] Reyes A, Wu M, McNulty NP, Rohwer FL, Gordon JI (2013). Gnotobiotic mouse model of phage-bacterial host dynamics in the human gut. Proc Natl Acad Sci U S A.

[201] Hsu BB, Gibson TE, Yeliseyev V, Liu Q, Lyon L, Bry L (2019). Dynamic modulation of the gut microbiota and metabolome by bacteriophages in a mouse model. Cell Host Microbe.

[202] Ott SJ, Waetzig GH, Rehman A, Moltzau-Anderson J, Bharti R, Grasis JA (2017). Efficacy of sterile fecal filtrate transfer for treating patients with Clostridium difficile infection. Gastroenterology.

[203] Citorik RJ, Mimee M, Lu TK (2014). Sequence-specific antimicrobials using efficiently delivered RNA-guided nucleases. Nature Biotechnol.

[204] Bikard D, Euler CW, Jiang W, Nussenzweig PM, Goldberg GW, Duportet X (2014). Exploiting CRISPR-Cas nucleases to produce sequence-specific antimicrobials. Nature Biotechnol.

[205] Isabella VM, Ha BN, Castillo MJ, Lubkowicz DJ, Rowe SE, Millet YA (2018). Development of a synthetic live bacterial therapeutic for the human metabolic disease phenylketonuria. Nature Biotechnol.

[206] Kurtz CB, Millet YA, Puurunen MK, Perreault M, Charbonneau MR, Isabella VM (2019). An engineered E. coli Nissle improves hyperammonemia and survival in mice and shows dose-dependent exposure in healthy humans. Sci Transl Med.

[207] Mimee M, Tucker AC, Voigt CA, Lu TK (2015). Programming a human commensal bacterium, Bacteroides thetaiotaomicron, to sense and respond to stimuli in the murine gut microbiota. Cell Syst.

[208] Lesterhuis WJ, Bosco A, Millward MJ, Small M, Nowak AK, Lake RA (2017). Dynamic versus static biomarkers in cancer immune checkpoint blockade: unravelling complexity. Nat Rev Drug Discov.

[209] Manichanh C, Reeder J, Gibert P, Varela E, Llopis M, Antolin M (2010). Reshaping the gut microbiome with bacterial transplantation and antibiotic intake. Genome Res.

[210] Adamberg S, Sumeri I, Uusna R, Ambalam P, Kondepudi KK, Adamberg K (2014). Survival and synergistic growth of mixed cultures of bifidobacteria and lactobacilli combined with prebiotic oligosaccharides in a gastrointestinal tract simulator. Microb Ecol Health Dis.

[211] Smillie CS, Smith MB, Friedman J, Cordero OX, David LA, Alm EJ (2011). Ecology drives a global network of gene exchange connecting the human microbiome. Nature.

[212] Ceroni F, Algar R, Stan GB, Ellis T (2015). Quantifying cellular capacity identifies gene expression designs with reduced burden. Nat Methods.

[213] Chan CT, Lee JW, Cameron DE, Bashor CJ, Collins JJ (2016). 'Deadman' and 'Passcode' microbial kill switches for bacterial containment. Nat Chem Biol.

[214] Milshteyn A, Colosimo DA, Brady SF (2018). Accessing bioactive natural products from the human microbiome. Cell Host Microbe.

[215] Chen H, Nwe PK, Yang Y, Rosen CE, Bielecka AA, Kuchroo M (2019). A forward chemical genetic screen reveals gut microbiota metabolites that modulate host physiology. Cell.

[216] Lange K, Buerger M, Stallmach A, Bruns T (2016). Effects of antibiotics on gut microbiota. Dig Dis.

[217] Sheth RU, Cabral V, Chen SP, Wang HH (2016). Manipulating bacterial communities by *in situ* microbiome engineering. Trends Genet.

